# Relay charge transport in thunderclouds and its role in lightning initiation

**DOI:** 10.1038/s41598-022-10722-x

**Published:** 2022-04-30

**Authors:** A. A. Syssoev, D. I. Iudin, F. D. Iudin, V. Yu. Klimashov, A. A. Emelyanov

**Affiliations:** 1 Laboratory of Nonlinear Physics of Natural Processes, Federal Research Center Institute of Applied Physics of the Russian Academy of Sciences, Nizhny Novgorod, Russia 603950; 2 Department of Medical Biophysics, Privolzhsky Research Medical University, Nizhny Novgorod, Russia 603005; 3 Geophysical Electrodynamics Department, Federal Research Center Institute of Applied Physics of the Russian Academy of Sciences, Nizhny Novgorod, Russia 603950

**Keywords:** Atmospheric science, Plasma physics, Natural hazards, Mathematics and computing

## Abstract

A new mechanism of charge transport inside a thundercloud is suggested and numerically investigated. The considered mechanism can be called “relay” because it is provided by a dynamical network of a relatively small amount of continuously decaying and arising conducting plasma formations. It manifests itself in two consecutive modes corresponding to pre-streamer and streamer/leader stages of thundercloud development. The first one is provided by dynamics of conducting ionic spots recently described by Iudin et al.^1^ that prepare conditions for initiation of positive streamers. The second mode relies on dynamical network of streamer/leader discharges and finally results in the formation of a compact well-conducting structure that bridges an area of strong electric field inside a thundercloud and can be associated with a lightning “seed”. The effectiveness of relay charge transport strongly depends on the relative proportion of conductive elements (plasma formations) and drastically increases in the field-dependent case.

## Introduction

Despite significant progress in the sphere of atmospheric electricity it is still unknown how a self-sustaining lightning channel forms inside a thundercloud^2^. The intrigue revolves around the fact that electric fields observed in thunderclouds have peak values of $(1{-}3) \cdot 10^{5}$ V/m [3, Table 3.2] (the maximum measured value is $4 \cdot 10^{5}$ V/m^4^), which is about an order of magnitude lower than the dielectric strength of air at typical lightning initiation altitudes. The lightning initiation scenario is likely to include the development of a streamer/leader network as an obligatory stage. It is noted by Dwyer and Uman^2^ that “In order to create and maintain a hot channel, the energy from the electrostatic field must be concentrated into a much smaller volume, where the heating occurs. This may happen via the creation of an extended streamer network, with many streamers feeding their current into a narrow channel ...”. An example of a complex hierarchical system of interacting channels at different stages of development (termed as unusual plasma formations or UPFs) arising inside an artificial cloud of charged water droplets was recently described by Kostinskiy et al.^5^. The authors state that “such formations can be an intermediate stage between virgin air (in the presence of water droplets) or initial low-conductivity streamer and a hot, self-propagating leader channel”. In this study, the role of collective effects in lightning initiation process is further analyzed. In particular, it is shown how a new type of charge transport, which can be termed as “relay”, first prepares conditions for an intracloud streamer network appearance and then results in a lightning leader formation.

Thanks to the great success of percolation theory, the problems of site and bound percolation on a cubic lattice became widely used in various fields of science^6^. It was established that if concentration of randomly spaced conducting links exceeds some critical value, which is called percolation threshold, there arise an “infinite” or percolating cluster which connects opposite borders of dielectric space and neutralises the applied electric field (causes a short circuit). At the same time, in many natural systems there may be a more interesting situation when a large-scale current appears between the borders of dielectric medium even in the subthreshold conditions, i.e. in the absence of any percolating conducting cluster. The case may be realized if a network of conducting links has time-dependent structure, i.e. some part of links continuously disappears and simultaneously reappears in other sites of the medium. In this case, charge transport along a large-scale electric field direction takes place because newly-formed clusters pick up charges left by the previous ones. This specific dynamics of the system development allows one to speak about *relay charge transport* or about *relay conductivity*. It turns out that, although there is no percolating cluster on a snapshot of the system, there forms a conducting chain in 4-D spatio-temporal continuum.

In a recent study by Iudin et al.^1^ it was shown that before the lightning initiation a thundercloud turns out to be seeded by ionic conductivity spots with characteristic scales of the order of 0.1–1 m and lifetimes in the range of 1–10 s. Typical conductivity of these plasma formations is many orders of magnitude bigger than that of the surrounding air ($10^{-10}{-}10^{-9}$ S/m versus $10^{-14}{-}10^{-13}$ S/m [3, Fig. 1.3]) which allows to consider them as conductors placed inside the dielectric medium. As these elevated conductivity regions continuously decay, mainly due to ions attachment to hydrometeors and turbulent air mixing, and appear due to corona discharges accompanying collisions (or nearly collisions) of hydrometeors, there must be the effect of relay conductivity in a thundercloud. At later stages ionic conductivity regions are replaced by streamer/leader systems. The latter contribute to relay charge transport as well, but are also capable to grow and are prone to merge with each other which makes their dynamics much more complex. In this study, the features of pre-streamer and streamer/leader-based modes of relay charge transport are analyzed and their role in lightning initiation process is discussed.

The content of the paper is as follows. Section “Pre-streamer mode of relay charge transport” is devoted to relay charge transport at the stage preceding streamers appearance. In subsection “Pre-streamer model description” a basic model which was developed to simulate the effect of relay conductivity in the framework of a simple cubic lattice for two different types of problem formulations is described. Subsection “Physical basis of the pre-streamer model” contains discussion of how the presented abstract numerical model can be coordinated with conditions of a real thundercloud. Subsection “Simulation results (pre-streamer stage)” presents the results of numerical experiments, the main of which are dependences of relay conductivity on the concentration of conductive elements (the filling factor) obtained for two different problem formulations, and discusses how the pre-streamer mode of relay charge transport provides multiscale electric field fluctuations eventually giving rise to positive streamers. In section “Streamer/leader-based mode of relay charge transport” an advanced self-organizing numerical model, which takes into account the ability of discharge structures to grow and merge with each other, is used to show how electrostatic interaction of numerous streamer/leader clusters makes their (streamer/leader-based) mode of relay charge transport more effective and finally results in a self-sustaining lightning leader formation. “Discussion” section shows the results of the study with an emphasis on comparing pre-streamer and streamer/leader-based modes of relay charge transport. In “Summary” section the main findings of the study are formulated.

## Pre-streamer mode of relay charge transport

### Pre-streamer model description

This subsection describes the features of a numerical model used to examine effective conductivity of the medium (times of electric field relaxation) due to the effect of relay charge transport preceding positive streamers initiation.

#### Problem formulation

**Figure 1:**
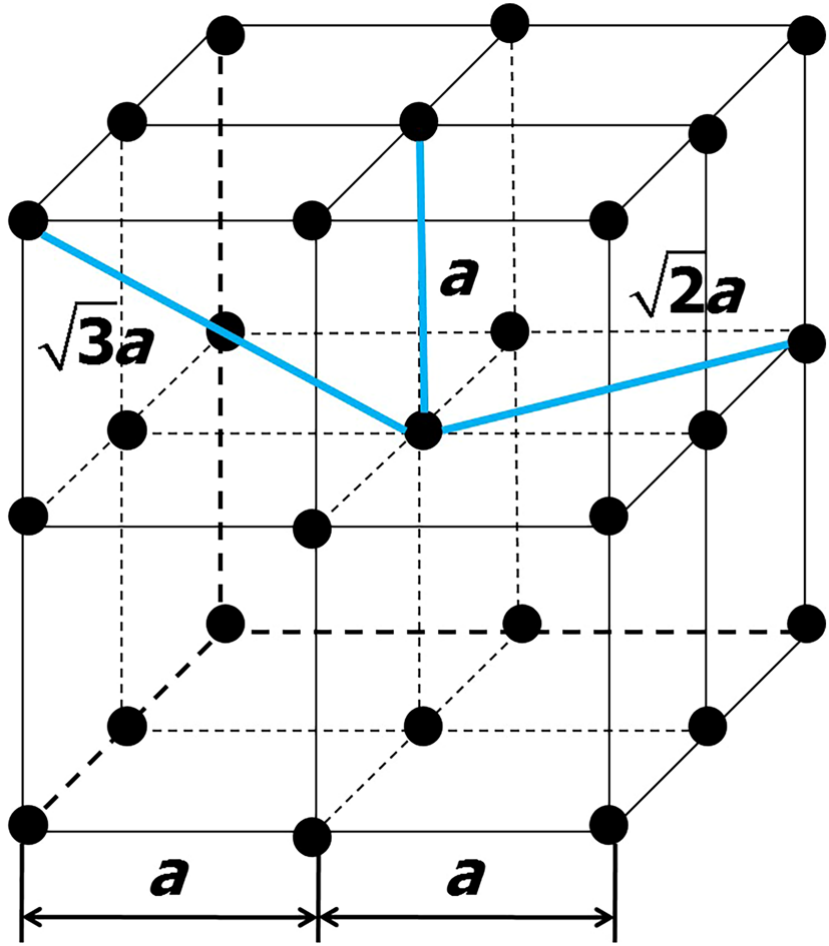
A fragment of 3-D simple cubic lattice showing three possible types of links between pairs of neighboring nodes. Each node can potentially be a source of 6, 12, and 8 links with the lengths of *a*, $\sqrt{2} a$, and $\sqrt{3} a$, respectively.

The model domain is a rectangular parallelepiped with dimensions of $30\times 30\times 60$ m$^3$. It is divided into identical cubic cells with an edge of $a = 1$ m, the vertices of which form a simple cubic lattice. A pair of neighboring nodes of space grid can be connected by a perfectly conducting link with possible lengths of *a*, $\sqrt{2} a$, and $\sqrt{3} a$ (see Fig. 1). There is a constant uniform vertically upward electric field ${\varvec{E}}_0$ with a conditional strength of 100 kV/m throughout the simulation domain which can be considered as an ambient one. It is assumed that before the simulation starts the model domain does not contain any conducting links and that all the space grid nodes are not charged.

In simulations the fraction of simultaneously existing conducting links or the filling factor *p* varies from 0.1% to 3.5% (with an increment of 0.1%) of the total number of links $N_{total}=720120$ available for the considered simple cubic lattice. Since the considered filling factors are relatively small, the system develops under the percolation threshold, which is 0.0497080(10)^7^ ($\approx$5%) for a simple cubic lattice with all the 26 links outgoing from each spatial grid node available.

**Figure 2:**
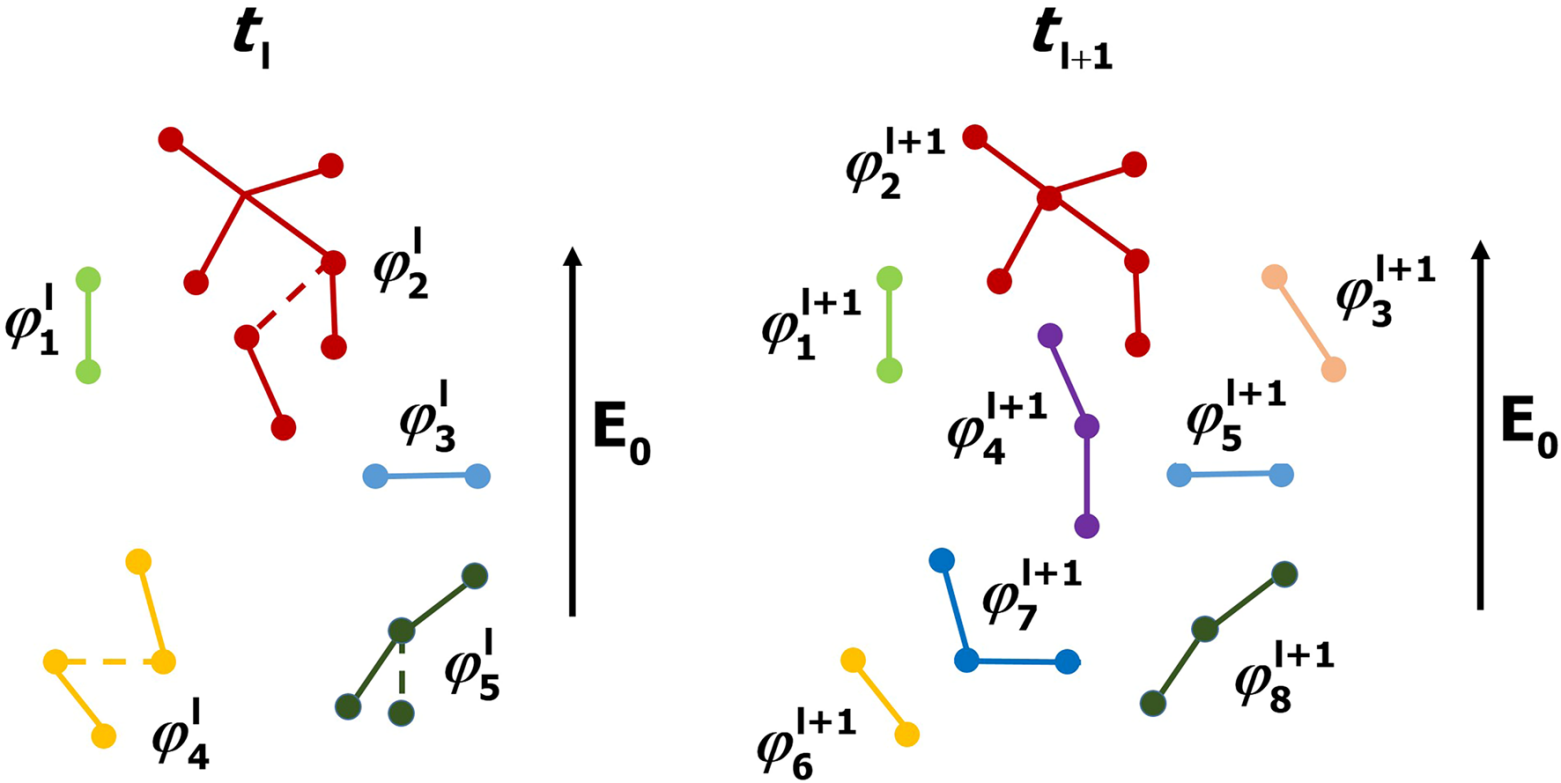
An example of alternating perfectly conducting clusters in an ambient electric field ${{\varvec{E}}}_0$ for the case of $u=20$% (each time iteration 3 out of 15 links disappear and reappear in random sites). Line segments and dots stand for links and spatial grid nodes, respectively. Individual equipotential clusters are shown in different colors. Model links which exist at the moment of time $t_l$ but disappear at the next time step $t_{l+1}$ are shown by dashed lines. The total number of links remains constant, while the number of clusters, as well as their electrical potentials and structures, can vary.

The network of model links is not static. Each time iteration a certain part of links disappears and simultaneously reappears in other free sites, i.e. between the neighboring space grid nodes which are not connected. A fraction of changing links is determined by the second parameter *u* (variability factor), the choice of which is discussed in the paragraph “The choice of variability factor”. At each model time step $\tau _m$, which depends on parameter *u* as discussed in the paragraph “General relations”, the choice of alternating links occurs randomly. An example of such a dynamical network of clusters is presented in Fig. 2.

#### Model equations

Perfectly conducting links arising in non-zero electric field cause redistribution of point space charges at the nodes of spatial grid they connect in order to equalize electric potentials of these nodes. The potential of the node with the radius-vector ${{\varvec{r}}}_k$ (with the number *k*) can be found as

1 $$\begin{aligned} \varphi ({\varvec{r}}_k)=\frac{1}{4\pi \varepsilon _0}\left( \sum _{i=1\text {, }i\ne k}^{N_{\text {tot}}-1}\frac{q_i}{|{\varvec{r}}_i-{\varvec{r}}_k|}+\frac{q_k}{(a/2)}\right) -({\varvec{E}}_0 \cdot {\varvec{r}}_k), \end{aligned}$$

where $\varepsilon _0=8.85 \cdot 10^{-12}$ F/m is permittivity of free space, $q_i$ is the point space charge located at the node with the radius-vector ${\varvec{r}}_i$, ($N_{\text {tot}}-1$) is the total number of point charges except $q_k$, *a*/2 = 0.5 m ($a=1$ m is the grid spacing) is the fictitious distance which is needed between the source and observation point to avoid singularity, and the last term (dot product of vectors ${\varvec{E}}_0$ and ${\varvec{r}}_k$) stands for potential provided by an ambient electric field at the point ${\varvec{r}}_k$. Note that the point charge self-contribution to electric potential in Eq. (1) is consistent with the general approach used in numerical techniques to avoid singularities (e.g., [8, Eq. 14.39 on p. 783]).

Each link can be either isolated or connected with other links. Hereinafter it is more convenient to describe the model algorithm in terms of clusters (note that isolated links can also be considered as minimal clusters consisting of only one element). As all the links are perfectly conducting, each cluster must be equipotential. Because of this, for any pair of nodes with indexes *i* and *j* belonging to the same cluster

2 $$\begin{aligned} \varphi ({\varvec{r}}_i)=\varphi ({\varvec{r}}_j). \end{aligned}$$

**Figure 3:**
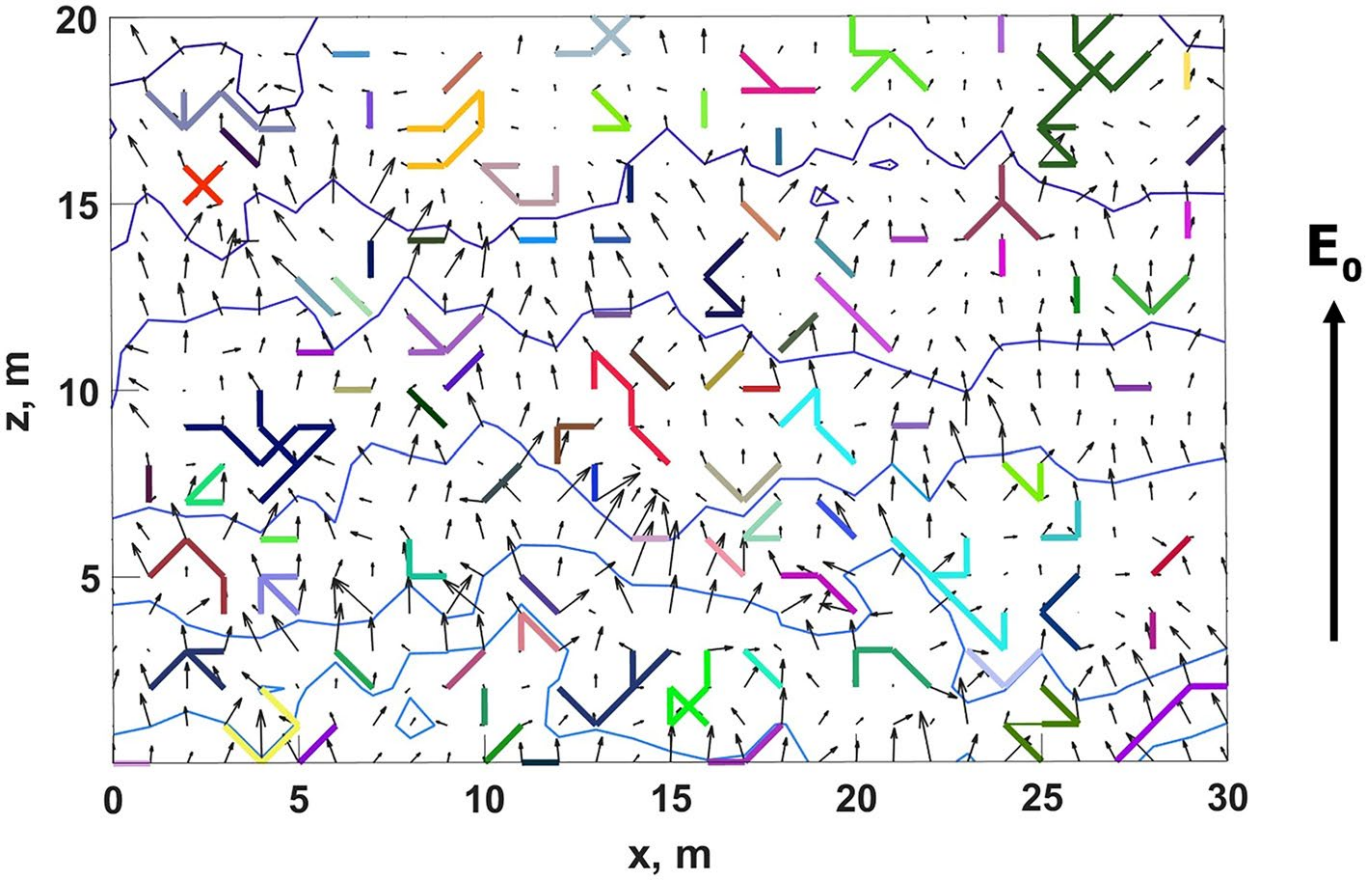
Illustration of the state of the model system for more demonstrative 2-D case. Individual equipotential clusters are shown in different colors. Blue lines and black arrows denote isolines of potential and electric field direction, respectively. At a given moment of time an ambient electric field ${\varvec{E}}_0$ is highly distorted by the preceding random dynamics of perfectly conducting clusters.

As there are no any external currents in the model, the total charge of the system is constant and equal to zero. Each time iteration point charges at the nodes of all the clusters are recalculated to ensure their equipotentiality. Although individual charges at the nodes belonging to a cluster can vary, the total charge of each cluster remains constant:

3 $$\begin{aligned} \sum _{i=1}^{N_{c}}q_i=\sum _{i=1}^{N_{c}}q'_i, \end{aligned}$$

where $N_{c}$ is the total number of nodes belonging to a cluster and $q'_i$ and $q_i$ are point charges of its *i*-th node before and after the recalculation procedure aimed at meeting the condition (2), respectively. Eqs. (2) and (3) allow one to find charges $q_i$ at all the nodes of any cluster at any moment of discrete model time. An illustrative example (a snapshot) of the 2-D model system state is shown in Fig. 3.

**Figure 4:**
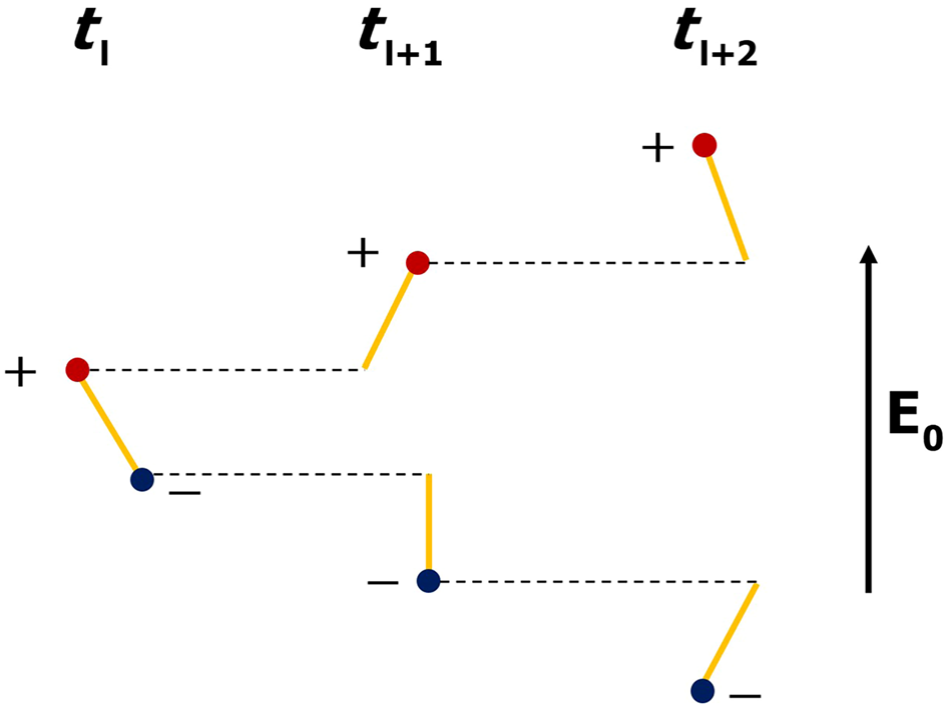
Simplified illustration of the scheme of relay charge transfer along an ambient electric field direction shown for three consecutive moments of discrete model time. Each model link arising between a pair of neighboring nodes creates dipole charge separation. Amplitudes of divided positive and negative charges, shown as red and blue dots, respectively, are assumed to be equal. The resulting current is directed along an ambient electric field vector.

Since each temporal iteration a fraction of model links disappears and reappears in random places, conducting clusters are not static but change their structures. Newly-formed links either create space charge separation (if they appear between previously uncharged nodes) or pick up space charges left by decayed clusters. They can also join already existing clusters or connect previously separated clusters, which is especially possible at relatively large values of the filling factor *p*. Stochastic dynamics of clusters gradually leads to a large-scale charge separation (see Fig. 4) and, as a consequence, to the reduction of a large-scale electric field in the interior of the system even in the absence of a conducting percolating cluster. Electric field evolution can be described by one of Maxwell’s equations with magnetic field being neglected:

4 $$\begin{aligned} \frac{\partial {\varvec{E}}}{\partial t}=-\frac{{\varvec{j}}}{\varepsilon _0}, \end{aligned}$$

where current density ${\varvec{j}}$ can be expressed via the effective conductivity $\sigma _{\text {eff}}$ as ${\varvec{j}}=\sigma _{\text {eff}}{\varvec{E}}$. Assuming that for fixed values of *p* and *u* relay conductivity is statistically constant in space and time, for the main (co-directed with ${\varvec{E}}_0$ vector) electric field component $E_z$ one has

5 $$\begin{aligned} E_z(t)=E_0 \cdot \exp \left( -\frac{\sigma _{\text {eff}}}{\varepsilon _0}t\right) . \end{aligned}$$

Relying on Eq. (5), in the study effective (relay) conductivity of the medium is defined as

6 $$\begin{aligned} \sigma _{\text {eff}}=\frac{\varepsilon _0}{\tau }, \end{aligned}$$

where $\tau$ is the time elapsed from the start of the simulation needed to fulfill the condition

7 $$\begin{aligned} E_{\text {eff}}=\frac{|U(z_{u})-U(z_{d})|}{z_u-z_d}\le E_0 \cdot e^{-1}. \end{aligned}$$

In Eq. (7) $U(z_u)$ and $U(z_d)$ are mean electric potentials at the levels of $z_u=48$ m and $z_d$ = 12 m which are used to find an averaged electric field magnitude in the inner part of the system. The usage of the middle part of the simulation domain is due to the well-known fact that electric field within a finite conducting system decreases in its interior and simultaneously increases at its boundaries (see Fig. 8). So, the central part of the model volume is considered to avoid the edge effect.

The system of linear equations to be solved at each discrete time step is presented in “Methods” section.

#### Links orientation

In the considered model, there are two ways to set orientations of links. The first (field-independent) one is to choose it randomly not getting attached to the local electric field direction. The second (field-dependent) possibility is to use the field-dependent probability distribution to increase the likeliness of model links orientation along the local electric field direction. In this case, the probability of a new link formation between a pair of neighboring nodes with the radius vectors ${\varvec{r}}_i$ and ${\varvec{r}}_j$ has the following (Weibull) form:

8 $$\begin{aligned} P(E_{r_i,r_j})=1-\text {exp}\left\{ -\left( \frac{E_{r_i,r_j}}{E_0}\right) ^2\right\} , \end{aligned}$$

where the electric field strength $\varvec{E}_{r_i,r_j}$ between these nodes can be found as

9 $$\begin{aligned} \varvec{E}_{r_i,r_j} = \frac{-(\varphi ({\varvec{r}}_i) - \varphi ({\varvec{r}}_j))\times({\varvec{r}}_i-{\varvec{r}}_j)}{|{\varvec{r}}_i - {\varvec{r}}_j|^2}. \end{aligned}$$

Note that the use of the field-dependent probability (8) ensures that the areas of locally increased electric field get filled with conductors first, which strongly influences the effective conductivity of the medium (see subsection “Simulation results (pre-streamer stage)”).

In simulations, both possibilities were tested to study to what extent this model feature influences the effective conductivity of the medium. In real systems the first variant is expected for relatively low (compared with the small-scale local electric field fluctuations) ambient electric field amplitudes, while the second one corresponds to relatively high values of the large-scale electric field. Since formation of the cloud charge structure is assumed to be accompanied by a large-scale electric field amplification [3, ch. 3], the field-depended case can be considered as a natural continuation of the field-independent one.

### Physical basis of the pre-streamer model

This subsection discusses how the described numerical model can be adopted to conditions of a real thundercloud.

#### General relations

To begin with, all characteristic timescales must be specified. Each conducting plasma formation (CPF) associated with a model link is characterized by a finite conductivity $\sigma$ and lifetime $\tau _l$. In order to consider real CPFs perfectly conducting, the condition $\tau _l\gg \varepsilon _0/\sigma$ must be fulfilled, which means that the lifetime of a CPF is sufficient for its polarization. One more characteristic timescale can be expressed via the spatio-temporal frequency of CPFs appearance $\nu$ and the volume of an active part of a thundercloud *V*. Indeed, the quantity $(\nu V)^{-1}$ presents the time needed for a single CPF formation.

In the model, it is assumed that the total number of CPFs *N* (or their relative fraction *p*) remains constant during the entire simulation time. It means that *uN* (*u* is the variability factor) CPFs must dissipate and appear during each model time step $\tau _m$:

10 $$\begin{aligned} \tau _m=\frac{uN}{\nu V}. \end{aligned}$$

On the other hand, assuming that the “age” distribution of CPFs is uniform on the interval $t\in [0; \tau _l]$, that is the total of $(\tau _m/\tau _l)N=uN$ “oldest” CPFs disappear during the time $\tau _m$, one can conclude that

11 $$\begin{aligned} \tau _m=u\tau _l. \end{aligned}$$

It follows from Eqs. (10) and (11) that

12 $$\begin{aligned} n_V=\tau _l\nu , \end{aligned}$$

where $n_V=N/V$ is the CPFs volumetric density. As $\tau _l\gg \varepsilon _0/\sigma$, the condition

13 $$\begin{aligned} n_V\gg \frac{\nu \varepsilon _0}{\sigma } \end{aligned}$$

must be fulfilled. The filling factor *p*, the most natural analogue of which is the relative share of space occupied by CPFs, can be defined as

14 $$\begin{aligned} p=\frac{N \cdot V_{CPF}}{V}=n_VV_{CPF}, \end{aligned}$$

where $V_{CPF}$ is a CPF volume. To verify the validity of relations (12) and (13) in conditions of a real thundercloud, one must first define the properties of CPFs.

#### Model link prototypes

In a recent study by Iudin et al.^1^, it was shown how an active part of a thundercloud gets seeded by ellipsoidal elevated ionic conductivity regions (EICRs) with characteristic sizes, lifetimes, and conductivities being of the order of 0.1–1 m, 1–10 s, and $10^{-10}{-}10^{-9}$ S/m, respectively. It was also justified that the influence of EICRs on electric field distribution becomes significant if the spatio-temporal frequency of their appearance exceeds quite a moderate level of 0.1 m$^{-3}$s$^{-1}$ (maximal possible values were estimated to be of the order of $10^2$ or even $10^3$ m$^{-3}$s$^{-1}$). Although conductivities of EICRs are not infinite (for comparison, typical conductivities of soil and virgin air at the sea level are $10^{-3}$ S/m and $10^{-14}$ S/m, respectively [3, Fig. 1.3]), their lifetimes are much bigger than the time of Maxwellian relaxation $\varepsilon _0/\sigma \approx 10^{-2}{-}10^{-1}$ s. So, the effect of the presence of EICRs on space charge and electric field distributions is approximately the same as if they were perfect conductors. Accordingly, EICRs can be considered as the first candidates for the role of perfectly conducting model links, at least at the pre-streamer stage of thundercloud development.

Substituting the values $\tau _l=1{-}10$ s and $\nu =0.1{-}100$ m$^{-3}$s$^{-1}$ into Eq. (12), one gets $n_V=0.1{-}10^3$ m$^{-3}$ which is orders of magnitude bigger than the lower threshold $\nu \varepsilon _0/\sigma$ from Eq. (13) calculated for $\sigma =10^{-10}{-}10^{-9}$ S/m. Taking into account that EICRs have a volume of the order of $10^{-3}{-}1$ m$^3$ and can overlap each other, an assumption of having 0.1–$10^3$ ellipsoidal EICRs in a volume of 1 m$^3$ seems to be reasonable. As an example, the values $\tau _l=1$ s, $\nu =0.1$–10 m$^{-3}$s$^{-1}$, and $n_V=$0.1–10 m$^{-3}$ can be considered. Provided that $n_V=$0.1/1/10 m$^{-3}$, the minimal (0.1%) and maximal (3.5%) values of *p* used in the study correspond to the scales of EICRs (neglecting the ellipticity of their form) being of 0.2/0.1/0.05 m and 0.7/0.3/0.15 m, respectively (see Eq. (14)), which is in a good agreement with 0.1-1 m range predicted by Iudin et al.^1^

In further calculations $\tau _l=1$ s is used, which is at the lower end of the range 1–10 s from the study^1^, at which EICRs were introduced. The minimum limit is chosen deliberately because it follows from general considerations that EICRs conductivity falls in time. It means that smaller lifetimes correspond to bigger conductivities (of the order of $10^{-9}$ S/m) which is closer to the concept of perfectly conducting links used in the model. As the model time step $\tau _m$ is directly proportional to the CPF lifetime $\tau _l$ (see Eq. (11)), predicted values of relay conductivity can, if necessary, be easily rescaled for other possible values of $\tau _l$ (see Eqs. (15) and (16)).

#### The choice of variability factor

Unlike the filling factor *p*, variability factor *u* must be considered as an adjustable model parameter. The closest to reality value of *u* must ensure that only one CPF disappears and reappears during a single time step $\tau _m$, i.e. $uN=1$ and $\tau _m=(\nu V)^{-1}$ (see Eq. (10)). It occurs because in a real situation new links appear sequentially and each of them changes the overall electric potential distribution before the appearance of a subsequent link. On the other hand, for a system consisting of thousands of simultaneously existing links the use of $u=N^{-1}$ requires unreasonably big computation time. Because of this, the used value of *u* is a compromise between the reasonableness of computation time and the correctness of the model results.

It follows from Eqs. (10) and (11) that the model time step $\tau _m$ is directly proportional to the variability factor *u*. As relay conductivity $\sigma _{\text {eff}}$ is inversely proportional to the time $\tau$ needed for electric field *e*-fold decrease (see Eq. (6)), for allowable values of *u* this time must be independent from *u*. In other words, for a fixed value of the filling factor *p* the number of model iterations $N_{it}$ needed to meet the condition (7) must be inversely proportional to *u*, because in this case $\tau =N_{it}\tau _m=N_{it}u\tau _l=const$. Calculations show that values of $N_{it}u$ keep nearly constant only for relatively small variability factors $u\le 10$%, which must be progressively smaller for increasing filling factors *p* (up to 0.05% for $p=$3.5%). So, to find correct times $\tau$ for each considered value of *p* it was checked that products $N_{it}u$ calculated for model realizations with the minimal and next to the minimal values of *u* differ no more than by 5%. To further ensure the calculation correctness, each value of time $\tau$ obtained for the minimal *u* was averaged over at least three model realizations before it is substituted in Eq. (6) to find $\sigma _{\text {eff}}$.

For a typical conductivity of EICRs being of $10^{-9}$ S/m, its electric field relaxation time $\varepsilon _0/\sigma$ is of the order of $10^{-2}$ s. So, to ensure that the model time step $\tau _m=u\tau _l\ge 10^{-2}$ s, for the chosen EICR life time $\tau _l=1$ s variability factor *u* should be no less than $10^{-2}$. In some numerical simulations variability factors smaller than that value were used, up to $5 \cdot 10^{-4}$ for the upper limit of *p*, especially in the field-dependent case. Although it means that in model realizations, for which the model time step was smaller than $\varepsilon _0/\sigma$, just reappeared links could not be considered as perfectly conducting, the inaccuracy not influencing simulation results dramatically. This is because in each case the entire simulation time $\tau$ was many times bigger than $\varepsilon _0/\sigma =10^{-2}$ s and because the relative share of links which “age” was smaller than $10^{-2}$ s did not exceed 1%. Note that it would be wrong to keep variability factor *u* at the level of $10^{-2}$ for the relatively big values of *p* because this would result in a big error in the model predictions of the field relaxation time $\tau$.

### Simulation results (pre-streamer stage)

**Figure 5:**
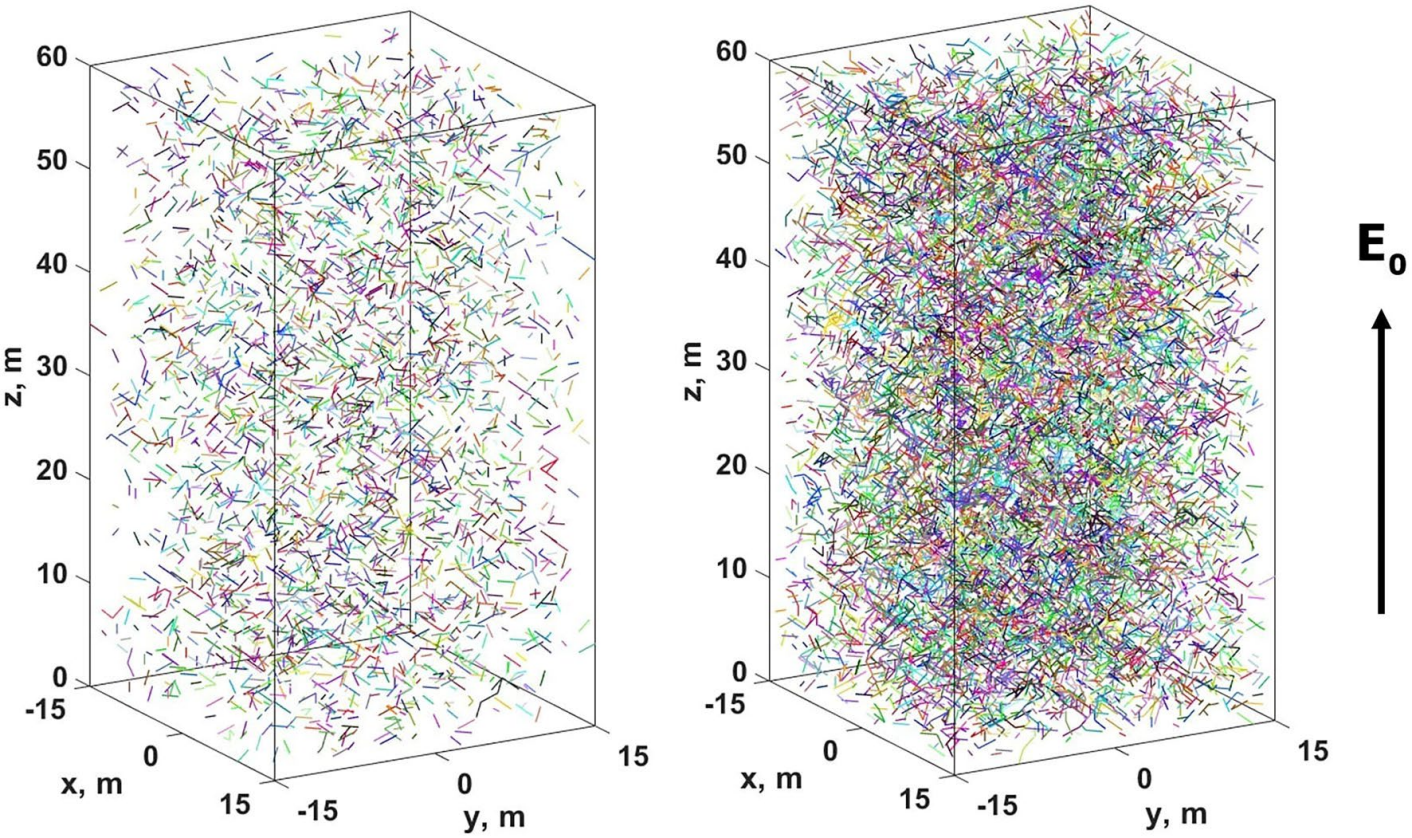
Snapshots of the model system for the field-independent case under the values of the filling factor *p* being 0.5% (on the left) and 2.0% (on the right). Individual equipotential clusters are shown in different colors.

As it was already noted, in numerical simulations of the pre-streamer stage of relay charge transport two different types of problem formulation were considered:

1. the choice of orientation of links is random and does not depend on the electric field (two examples of the system state corresponding to this case are shown in Fig. 5);
2. the choice of orientation of links is determined by the field-dependent Eq. (8).

**Figure 6:**
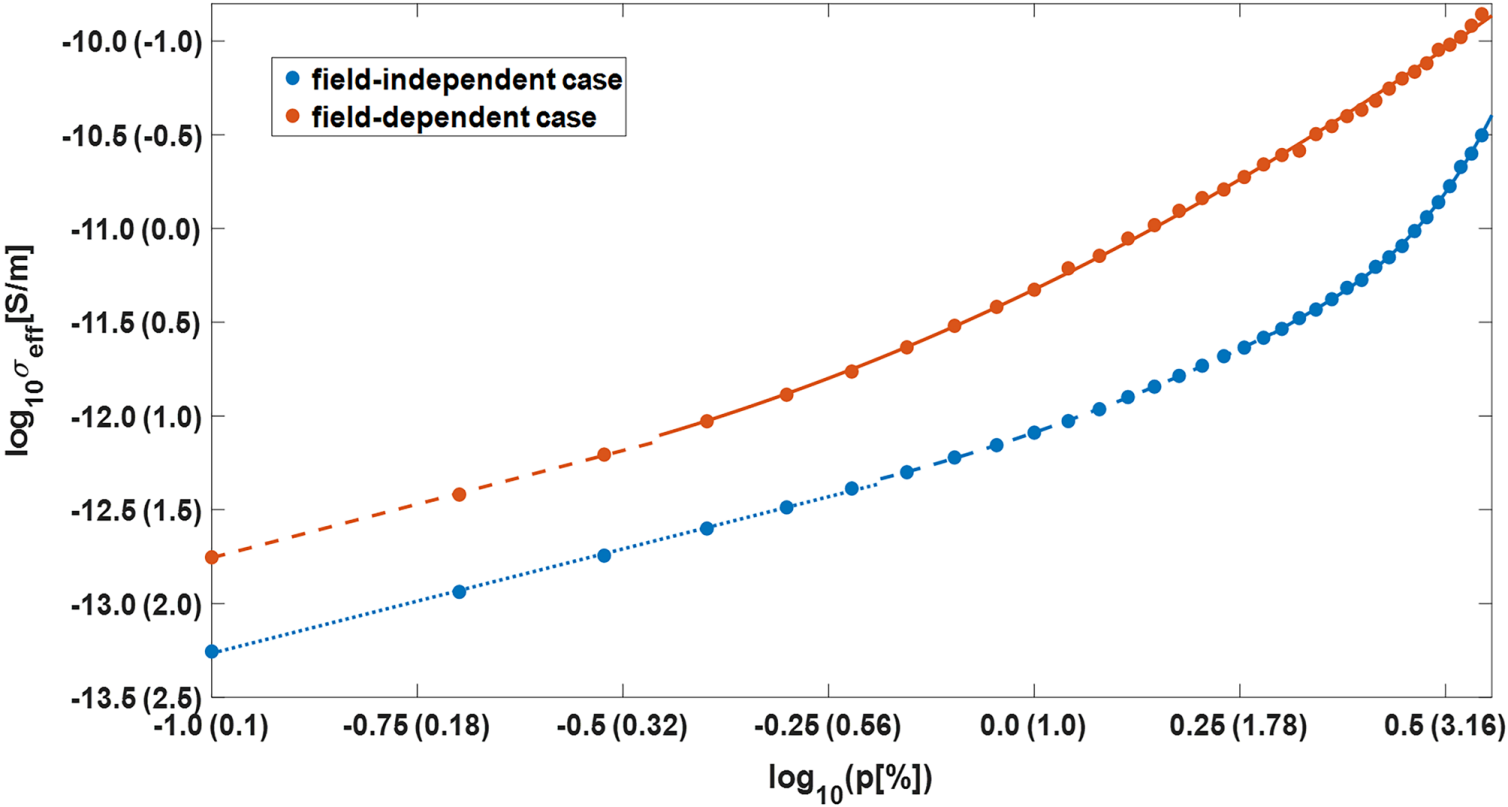
Dependence curves (16) of effective (relay) conductivity $\sigma _{\text {eff}}$ on the filling factor *p* for field-dependent and field-independent cases shown in bi-logarithmic axes with a model link lifetime $\tau _l$ assumed to be 1 s (see paragraph “Model link prototypes”). On horizontal and vertical axes additional values shown in the parentheses stand for filling factors *p* expressed in percents and decimal logarithms of the corresponding electric field relaxation times $\tau =\varepsilon _0/\sigma _{\text {eff}}$ expressed in seconds (with permittivity of free space $\varepsilon _0$ rounded to $10^{-11}$ F/m), respectively.

One of the main aims of the study is to find out how relay conductivity of the medium $\sigma _{\text {eff}}$ depends on the filling factor *p*. It is supposed that the sought dependence has the following form:

15 $$\begin{aligned} \sigma _{\text {eff}}[\text {S/m}]=\frac{1\text { F/m}}{\tau _l[\text {s}]}10^{\alpha _0+\sum _{i=1}^{3}\alpha _ilog^i_{10}p}, \end{aligned}$$

where $\alpha _i$ are some fitting parameters which can vary from one range of *p* to another. The results of numerical analysis for the two considered cases are presented in Fig. 6 and have the following forms:

16 $$\begin{aligned} \begin{array}{l} \sigma _{\text {eff}}[\text {S/m}]\text {(field-independent case)}=\frac{1\text { F/m}}{\tau _l[\text {s}]} \left\{ \begin{array}{l} 10^{-12.15+1.11\log _{10}p},\text { }p\le 0.6\%\\ 10^{-12.09+1.52\log _{10}p+1.06\log _{10}^2p},\text { }0.6\%< p\le 1.9\%\\ 10^{-13.30+12.29\log _{10}p-31.54\log _{10}^2p+33.85\log _{10}^3p},\text { } p>1.9\%. \end{array}\right. \\ \\ \sigma _{\text {eff}}[\text {S/m}]\text {(field-dependent case)}=\frac{1\text { F/m}}{\tau _l[\text {s}]} \left\{ \begin{array}{l} 10^{-11.61+1.14\log _{10}p},\text { }p\le 0.3\%\\ 10^{-11.32+2.12\log _{10}p+0.91\log _{10}^2p},\text { }p>0.3\%. \end{array}\right. \end{array} \end{aligned}$$

It is seen in Fig. 6 that the chosen form of dependence (15) fits the simulation results well. It follows from general considerations that linear (in the log-log space) parts of Eqs. (16) can be extrapolated to the area of smaller filling factors because the impact of cluster-cluster aggregation (see the next paragraph) is negligible for $p\lesssim$0.3%, i.e. there should not be fundamental changes in the system dynamics. For $p\ge$3.5% the rate of relay conductivity growth must increase dramatically because it is known from the percolation theory that $\sigma _{\text {eff}}$ strives to infinity in the percolation threshold ($p_c\approx$5%) vicinity. In the field-dependent case, for which channels orientation is determined by Eq. (8), effective conductivity is expectedly bigger than that in the field-independent case with a totally random orientation of links. This is because the use of Eq. (8) ensures that at least at the beginning of the simulation the vast majority of links (all of them at the first iteration) is oriented along an ambient electric field. Another factor is that in the field-dependent case the probability (8) of links appearance inside the gaps between closely spaced (assumed to be separated along a large-scale electric field direction) polarized clusters, where electric field is locally amplified, is increased. As a result, there is a tendency to clusters association, which can be considered as a kind of self-organization, that makes charge transport more effective.

Forms of Eqs. (16), which are used to fit simulation results, evidence that for filling factors *p* exceeding a conditional threshold of about 1% the rate of relay conductivity growth accelerates rapidly. This is because, in accordance with the percolation theory, characteristic scale of a typical cluster is proportional to $|p-p_c|^{-\xi }$, where $p_c= 0.0497080(10)$^7^ is the percolation threshold and $\xi$ is the critical exponent which is equal to 0.875^6^ for the considered 3-D case. It means that for relatively big values of *p* not only the relative share of conductive links by itself, but also the factor of clusters enlargement becomes significant. Indeed, this is more effective to transfer charge by connecting (equalizing potentials of) relatively large clusters than just by picking up point space charges by means of separated single links. Note that for relatively big filling factors *p* the predicted values of relay conductivity are many orders of magnitude bigger compared to a virgin air at the cloud altitude (about $10^{-13}$ S/m [3, Fig. 1.3]), especially for the field-dependent case. This finding may be especially important for the intracloud air, which conductivity is expected to be at least 2 orders of magnitude lower compared with that outside the cloud at the same height because of smaller concentration of ions in the interior region of the cloud^9^. This is apparently caused by the ions attachment to the water droplets (study^9^ did not consider the presence of ice in the cloud).

**Figure 7:**
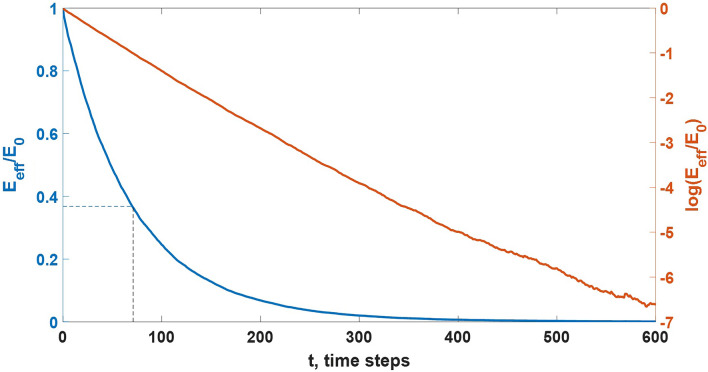
Dependence curves of normalized averaged electric field $E_{\text {eff}}$ calculated with the use of Eq. (7) (blue line) and its natural logarithm (orange line) vs time (expressed in time steps) for the field-independent case under $p=0.1$% and $u=10$% (a model time step $\tau _m=u\tau _l=$0.1 s for the considered lifetime of a link $\tau _l=1$ s). Dotted lines indicate the point ($\tau$, $e^{-1}$) which is used to find relay conductivity $\sigma _{\text {eff}}$ from Eq. (6).

Let’s discuss an example of model realization obtained for field-independent case under $p=0.1$% and $u=10$% to show in detail how electric field inside simulation domain evolves in time. Fig. 7 presents temporal evolution of the averaged electric field $E_{\text {eff}}$ (see Eq. (7)) in the central part of the model volume. It is clearly seen that $E_{\text {eff}}$ falls exponentially in time, as it was predicted by Eq. (5) for the case of $\sigma _{\text {eff}}=const$. Insignificant deviations from the basic form (5), manifestations of which are seen in logarithmic scale at long times, are because local electric field fluctuations (see Fig. 9) become more significant on the background of nearly zero average level.

**Figure 8:**
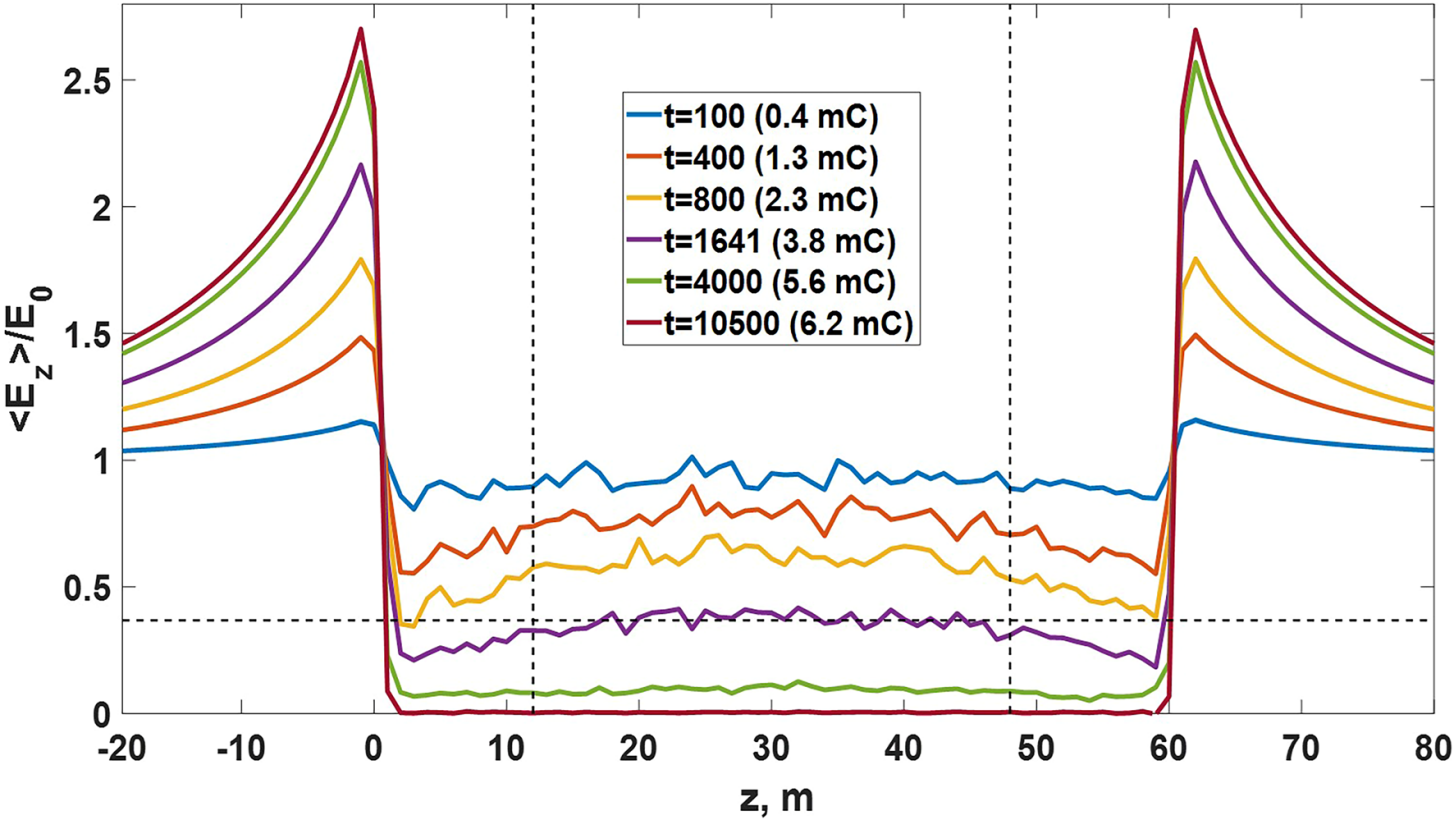
Distribution curves of normalized electric field projections $E_z$ averaged over *x* and *y* axes inside ($0\le z \le 60$ m) and outside ($z<0$ m and $z>60$ m) the simulation domain at different moments of time (expressed in model time steps) for the same case as in Fig. 7. Horizontal dotted line indicates the level of $e^{-1}$, while the vertical ones denote applicates $z_b=12$ m and $z_u=48$ m used in Eq. (7) to find an averaged field $E_{\text {eff}}$ inside the middle part of the simulation domain. For each electric field distribution, the total charge divided by relay charge transport is given in the legend.

In numerical simulations it is assumed that there are no conducting links beyond the model volume, i.e. effective conductivity is nonzero only inside it. Due to this electric field decrement in the middle part of the simulation domain, where $E_{\text {eff}}$ goes to zero, is accompanied by its simultaneous increment at the pair of boundaries of a conducting area that are perpendicular to an ambient electric field ${\varvec{E}}_0$ direction. It follows from Figs. 7 and 8 that the volume, in which relay charge transport develops, is similar to the conductor with a uniform conductivity $\sigma _{\text {eff}}$. Each electric field distribution curve shown in Fig. 8 has a local maximum at the central area ($z\approx$30 m), which results from the finiteness of the model volume. Since it is assumed that the medium beyond the simulation domain boundaries is non-conducting, space charge separated due to relay conductivity accumulates at the faces of the model parallelepiped, for which $z=0$ m and $z=60$ m, while at the central part of the model volume the average charge density remains zero. Such charge distribution provides a slight arch in shape of normalized electric field vs *z*. The discussed effect vanishes if the simulation domain dimensions strive to infinity.

**Figure 9:**
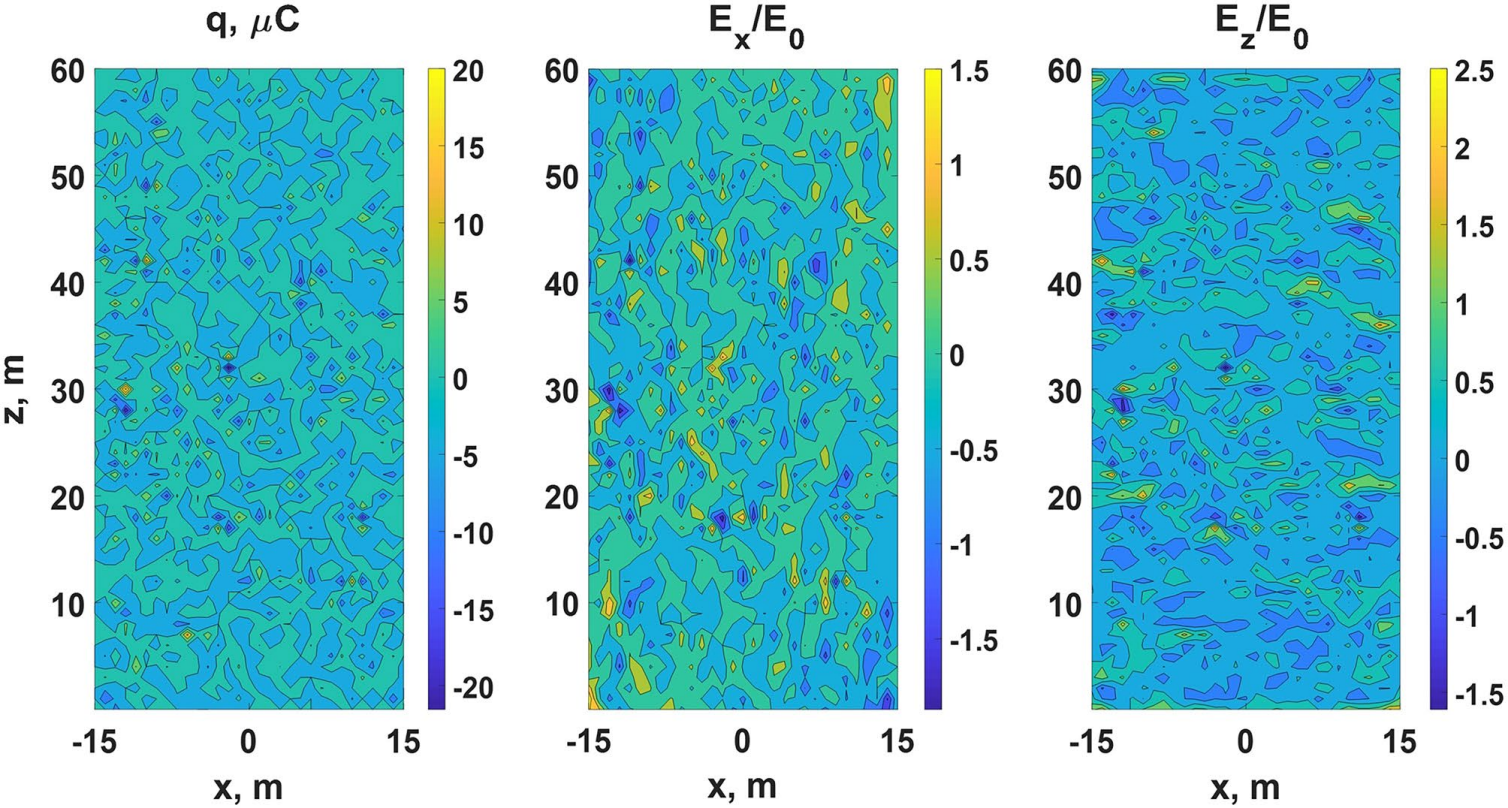
A snapshot of space charge (the left panel) and normalized $E_x$ and $E_z$ electric field projections (the middle and the right panels, respectively) distributions in the *x*-*z* plane at the final moment of discrete time at which $E_{\text {eff}}\approx E_0e^{-1}$ obtained for the field-independent case under $p=3$%. A system of alternating conductive clusters (polarized EICRs), concentrating positive and negative charges at their tips, provides space charge heterogeneities and, as a consequence, multiscale electric field fluctuations (see also Fig. 3), the amplitudes of which can easily exceed the level of an ambient electric field $E_0=10^5$ V/m. Some of these areas of strong electric field can become the sources of positive streamers.

It follows from Fig. 8 that relay charge transport results in electric field increase at two opposite (those which are perpendicular to ${\varvec{E}}_0$ vector) outer boundaries of a volume filled with EICRs (or any other CPFs). As real spatial distribution of EICRs cannot be homogeneous, there must be complex time-dependent multiscale electric field relief inside an active part of a thundercloud (see Fig. 9) with randomly alternating areas of locally increased and decreased electric field. Since the spatio-temporal frequency of EICRs generation gradually increases (see “Discussion” section), the amplitudes of electric field fluctuations must rise as well. At some moment of time local electric field fluctuations begin to exceed the level of positive streamers initiation, and the cloud volume gets filled with the “gas” of streamers. Since streamers (as opposed to EICRs) are capable to grow, the further dynamics of cloud charge transport cannot be reproduced in the framework of the basic (pre-streamer) model. In the next section, an advanced model is presented that is aimed to show how multiple streamer systems, arising as a result of described EICRs dynamics, contribute to another type of relay charge transport, which can conditionally be termed as “streamer/leader-based”. The second mode, although with several significant modifications, repeats the described relay mechanism on progressively larger spatial scales. It will be shown that the capability of self-organizing streamer/leader systems to grow and merge with each other qualitatively changes the way of charge transport strongly increasing its effectiveness.

## Streamer/leader-based mode of relay charge transport

In this section, an advanced numerical model of discharge development is presented which is applied to reproduce the relay charge transport features at the streamer/leader stage. The fundamental difference between the two model approaches is that, as opposed to EICRs, streamer/leader systems are able to grow, which facilitates their merger caused by electrostatic attraction.

### Discharge model description

In this subsection the features of the advanced model that is used to study the streamer/leader-based mode of relay charge transport are discussed. The applied approach is close to that employed in studies^10–12^ with the specific features thoroughly described below.

#### Problem formulation

The model domain is a rectangular parallelepiped with dimensions of $60\times 60\times 120$ m$^3$. It is divided into identical cubic cells with an edge of $a = 1$ m, which coincides with the characteristic length of streamer discharges arising from EICR poles^1^, and located at an altitude of 6 km above sea level which is typical of -CG lightning discharge initiation occurring between the main negative and the lower positive (if any) charge regions^13^. The vertices of the model space grid cells form a simple cubic lattice, at the nodes of which the model discharge trees develop. The mean channel length averaged over 26 possible directions of growth is $L=(6+12\sqrt{(}2)+8\sqrt{(}3))a/26\approx 1.42$ m. Taking into account that the minimum propagation speed of streamers in air typical of the initial stage of discharge development, at which electric fields are relatively low, $v_{min}\approx 2 \cdot 10^5$ m/s^14^, the model time step, at which the discharge tree morphology is updated, is set to $L/v_{min}\approx 7.1$ $\mu$s. The model does not consider the dependence of the streamer speed on air humidity and other factors discussed in study^15^. Discharge evolves in a uniform upward directed electric field ${\varvec{E}}_0$ with a strength of 65 kV/m which is lower than [3, Table 3.2] or at the lower boundary of [2, Table 3.1] maximum experimentally observed intracloud electric fields.

#### Electric potential and electric field calculation

Electric potential $\varphi$, produced by both constant electric field ${\varvec{E}}_0$ and point charges located at the grid nodes of the computational domain, and electric field between a pair of neighboring nodes can be found via the same formulas (1) and (9). When calculating electric potential, images of point space charges in highly remote ground surface were neglected.

#### Seeding positive streamers origin

At the first time iteration the system consists of 80 randomly spaced seeding positive streamer channels. Since model links can die off (see paragraph “Channels decay”), at the following iterations the total number of individual streamer structures is artificially maintained to be no less than 80 (additional streamer channels are added if necessary). This corresponds to spatio-temporal frequencies of new positive streamer links appearance $\nu$ varying from 0 to 26 m$^{-3}$s$^{-1}$. Directions of seeding positive streamers growth coincide with that of local electric fields (specified next). It is assumed that the appearance of these seeding links is associated with the small-scale (tens of centimeters) electric field fluctuations described in the studies^1, 16^, the source of which is the cloud ions dynamics. Since the model grid spacing of 1 m does not allow to describe these field bursts properly, they can be considered as manifestations of an external subgrid effect. So, the local electric field, in which (and along which) primary positive streamers originate, is a vector sum of electric field (9) provided by point space charges located at the nodes of model space grid and a randomly oriented small-scale intensification with an amplitude of $E_{pth}^+=2.45 \cdot 10^5$ V/m. The latter is the minimal field needed to support positive streamers propagation scaled to the considered 6 km altitude (at the ground level it is approximately equal to $5 \cdot 10^5$ V/m^14^).

#### Channels initiation and growth

The basic element of the advanced model is a link connecting a pair of neighboring space grid nodes. Each channel segment is a virtual object (a rectangular slab or a thin cylinder) with the only parameter that depends on the cross-sectional area being its conductivity (see study^11^ for more details). Each model link can grow by emanating new ones from its tips. A newly-formed model link can be considered as a bunch of co-directed streamers of the same polarity. Channel increment, that is the appearance of a new channel segment between a previously disconnected pair of neighboring space grid nodes with the radius vectors ${\varvec{r}}_i$ and ${\varvec{r}}_j$, is considered to be a probabilistic process. The corresponding probability is described by the same function (8), which is used in the basic model to determine orientations of links, but with another characteristic fields $E_{pth}^\pm$ instead of $E_0$. These fields are propagation thresholds for positive and negative streamers scaled to the considered 6 km altitude. In accordance with the well-known asymmetry^17^, $E_{pth}^-=2E_{pth}^+=4.9 \cdot 10^5$ V/m. It is assumed that any node belonging to a positive or a negative link can generate new streamer links of the same polarity, while the source nodes (from which bipolar discharge structures originate) can produce links of both polarities. Positive and negative channels grow towards and against the electric field direction, respectively. The use of asymmetry factor of 2 between threshold propagation fields of positive and negative streamers is discussed in a modeling study^11^. Further, the model considers the possibility of initiation of new streamer links under the influence of electric field intensification at the front of a sufficiently long well-polarized leader channel, which should be distinguished from the seeding channels appearance discussed in the previous paragraph. This process is also described by the formula (8) with characteristic field $E_{ith}$ instead of $E_0$, where $E_{ith}$ is the threshold electric field needed for positive streamers initiation from the previously free node that can be associated with a space stem (see study^11^ for more details). The value $E_{ith}=1.47 \cdot 10^6$ V/m is used for initiation (formation between a pair of nodes which were previously free from any discharge formations) of a positive streamer link. This value is the dielectric strength of air $E_b\approx 3 \cdot 10^6$ V/(m $\cdot$ atm)^14^ scaled to the 6 km altitude, which corresponds to positive streamers due to the fact that in natural conditions they always appear before negative ones. Note that the use of thresholdless probability formula (8) assumes that the real small-scale field at the tip of a growing streamer/leader channel is bigger than that calculated with the use of Eq. (9) because of local (subgrid) effects that cannot be taken into account in the framework of a model with a relatively large grid spacing of 1 m.

#### Channel parameters evolution

Channel conductivity $\sigma$ varies by orders of magnitude as its temperature increases due to Joule heating and decreases due to cooling processes. In this model, a simple empirical parametrization of the channel conductivity evolution is used, which is given by the following equation (also used in studies^10–12^):

17 $$\begin{aligned} \frac{\partial \sigma }{\partial t} = (\eta E^2 - \beta )\sigma , \end{aligned}$$

where $\eta =3 \cdot 10^{-5}$ m$^2$V$^{-2}$s$^{-1}$ and $\beta =3 \cdot 10^3$ s$^{-1}$ are parameters representing the rates of channel heating and cooling, respectively. An initial value of the newly-formed channel conductivity $\sigma _0$ is set to $10^{-9}$ S/m. Further, following Syssoev et al.^11^, it is assumed that the conditional threshold conductivity separating streamer channels from the leader ones is 1 S/m.

Electric field along a link gradually relaxes from the pre-breakdown value to the hot-channel one because of potential equalization by currents flowing through all the discharge tree channels^10–12^. For each link joining a pair of space grid nodes with radius vectors ${\varvec{r}}_i$ and ${\varvec{r}}_j$ this current is found from Ohm’s law as

18 $$\begin{aligned} I_{r_i,r_j} = \sigma _{r_i,r_j} \pi r^2 E_{r_i,r_j}, \end{aligned}$$

where radii *r* of all the channels are assumed to be equal to 1 mm.

The field-relaxation currents given by Eq. (18) lead to polarization of the entire conducting discharge tree in the overall electric field and to charge accumulation at its tips. This means that electric field decreases in the interior of the discharge structure with its simultaneous intensification at the discharge tree periphery^10–12^. To ensure the stability of the used numerical scheme, electric potential distribution $\varphi ({\varvec{r}})$ and channel conductivities $\sigma _{r_i,r_j}$ and currents $I_{r_i,r_j}$, given by Eqs. (1), (17), and (18), respectively, are recalculated each $\Delta t=14.2$ ns, which is 500 times smaller than the model time step $\tau _m=7.1$ $\mu$s.

#### Channels decay

Some discharge tree branches may stop growing and decay. If a peripheral channel (regardless of its conductivity) does not generate at least one new link during the model time step, it dies off. The charge previously transported to the node corresponding to the decayed branch tip (or to both tips if the decaying structure is a single floating link) remains “frozen” at that node (or nodes), because of both a low conductivity of the medium and a relatively small (compared to the charge relaxation duration) time needed for the discharge development (921 $\mu$s). These “frozen” charges contribute to the formation of the leader corona sheath (see, for example [14, Fig. 2.2] and [11, Fig. 5]).

Note that in this model channel extension and decay may be occurring simultaneously in different parts of the discharge tree and that the number of links originating from the same parent node is limited to 26 (see Fig. 1).

#### Discharge structures aggregation

If at some moment of discrete time a newly-formed streamer channel connects two nearly spaced but previously separated discharge structures, they merge. Clusterization of discharge structures is accompanied by potential equalization currents (see studies^11, 12^) that redistribute charges at the nodes of a joint system of conducting channels similar to how it occurs in the basic model but, because of finite streamer/leader links conductivity, for more than one time iteration. In this model, the self-inductive electric field provided by an intensive current pulse accompanying the merger of a pair of leader structures is not taken into account as it was shown by Syssoev et al.^12^ that it can be neglected compared to the electrostatic component.

#### Comparison with the basic model

Although both the basic (pre-streamer, see subsection “Pre-streamer model description”) and advanced models describe charge transport by means of a dynamical network of conducting channels, the latter one is more complex and can be considered as an extension of the basic approach. The advanced (lightning initiation) model additionally takes into consideration the asymmetry between characteristic fields needed to support positive and negative streamer development, evolution of electrical parameters (conductivity and internal electric field) of channels, and the possibility of simultaneous growth and decay of discharge tree branches. It also has no limitations on the total number of channels, except one concerning the minimal amount of seeding positive streamer structures, which is important only at the beginning of the simulation. These features allow an advanced model to reflect the self-consistent connection between the changes of electric field distribution and discharge system evolution (see also studies^10–12, 18^).

### Simulation results (streamer/leader-based stage)

**Figure 10:**
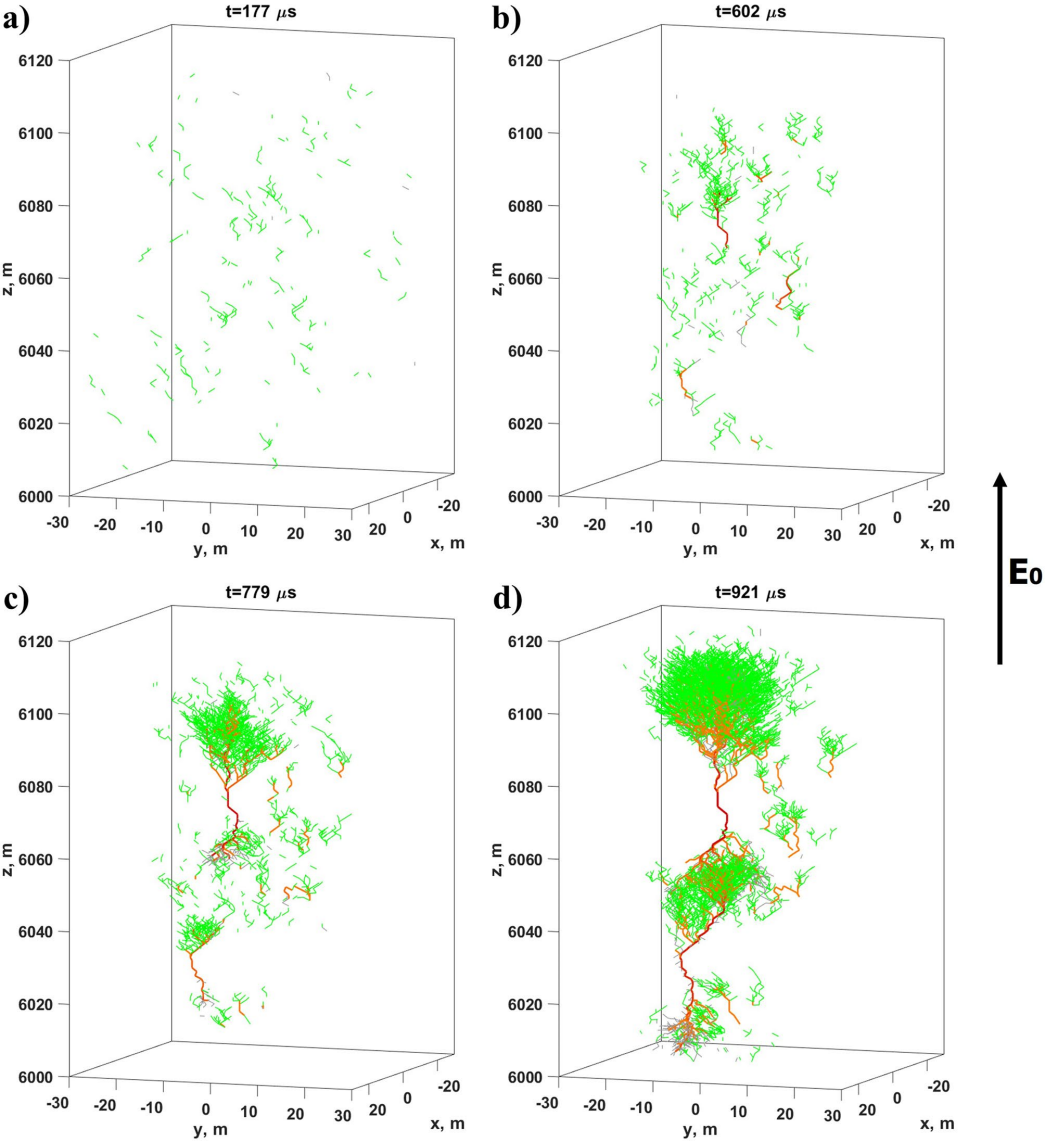
Visualization of different stages of lightning leader formation via coalescence of multiple simultaneously developing bipolar streamer/leader systems. Positive and negative streamer channels are shown in green and gray, respectively. Red channels indicate hot leader sections with conductivities exceeding 1 S/m (darker shades correspond to higher values of conductivity).

In the considered case, an external vertically oriented electric field ${\varvec{E}}_0$, which is set to 65 kV/m, is significantly lower than the propagation threshold of positive streamers which is equal to 245 kV/m at the considered 6 km altitude. Because of this at the beginning of the simulation the ambient electric field cannot support the growth of the vast majority of appearing positive streamers (according to Eq. (8), probabilities of extension of positive and negative streamer links in an ambient electric field $E_0$=65 kV/m are only 6.8% and 1.7%, respectively). In this connection, first generations of positive streamers, the birth of which does not depend on the field (9) and is considered to be related to the small-scale electric field fluctuations arising as a result of the negative ions dynamics^1, 16^ (see paragraph “Seeding positive streamers origin”), mostly decay. In ambient electric fields, the amplitudes of which exceed the level of about 55 kV/m (the value established via multiple numerical experiments), a small amount of initial streamers can generate new links from their tips and, thus, has a chance to avoid extinction. These “successful” seeding streamers first develop into bipolar streamer systems and then into bipolar leader channels (see Fig. 10(b,c,d)). At the leader stage the discharge tree can easily support its further propagation by itself even in a small external electric field due to polarization effect (see Fig. 10(c,d)). The most intensive processes at this phase are connected with the multiple junctions arising between bipolar streamer/leader systems at different stages of their development (the most potent connection occurred just before the stage (d) in Fig. 10). It is important that throughout the discharge development the rate of its evolution rapidly increases because of strong electric field enhancement at the tips of long well-conducting leader channels.

**Figure 11:**
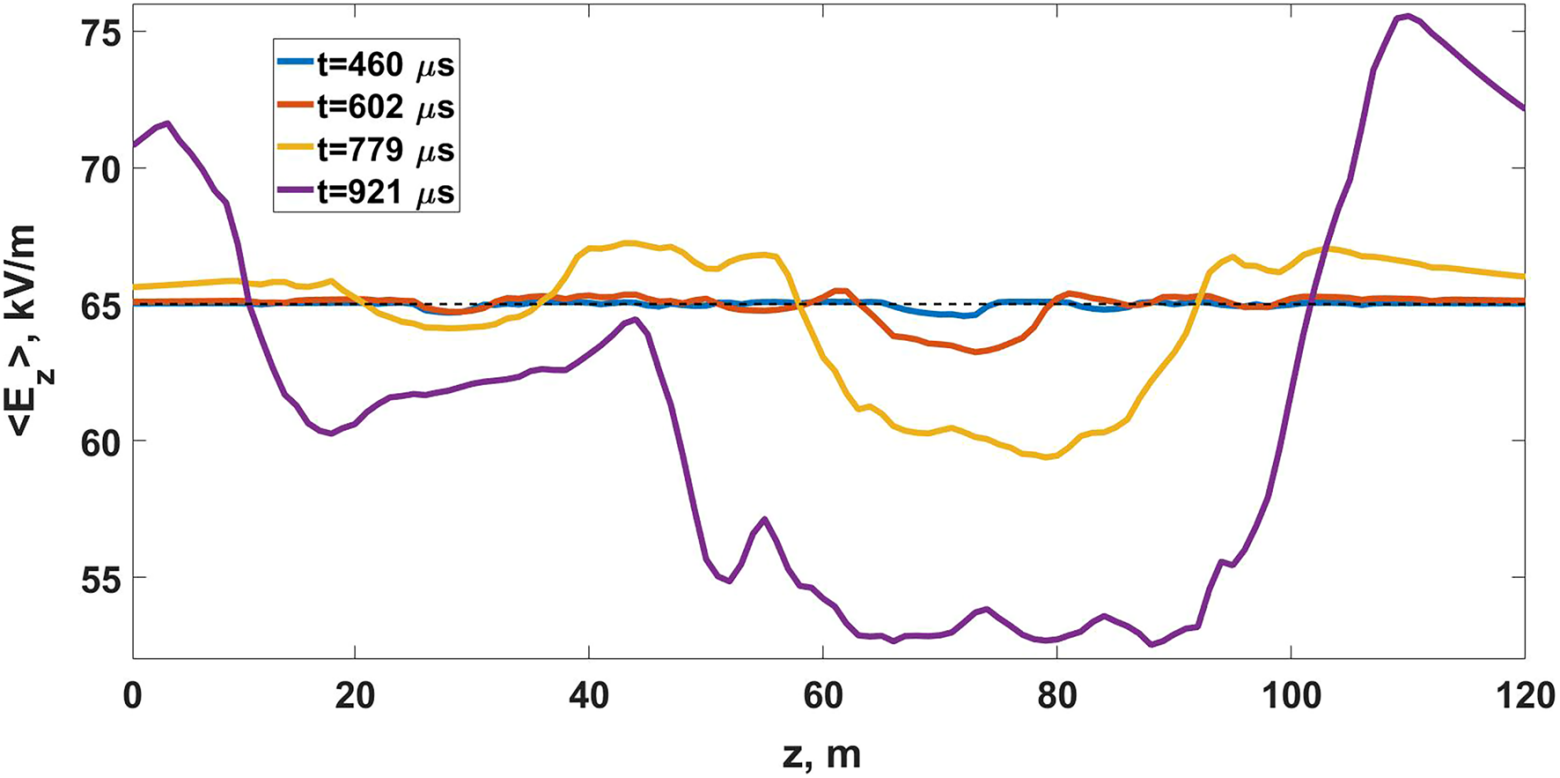
Dependence curves of electric field *z*-projections averaged over the area of $-20\le x[\text {m}]\le 20$ and $-20\le y[\text {m}]\le 10$, which includes the vast majority of model links (see Fig. 10) during the entire simulation time, shown for several moments of time. Black dashed line denotes the level of an ambient electric field $E_0=65$ kV/m.

At the developed stage simulated channel system change initial electric field distribution in a very effective way. Unlike the more homogeneous case described in subsection “Simulation results (pre-streamer stage)”, quasi-electrostatic interaction of well-polarized bipolar leader channels gradually leads to their merger: there arise a compact (fractal) network of well-conducting channels which quickly pushes the electric field from its interior to its periphery but does not noticeably affect electric field inside the surrounding area. It follows from Fig. 11 that $E_z$ electric field component averaged over the volume including the vast majority of model links does not vary significantly.

**Figure 12:**
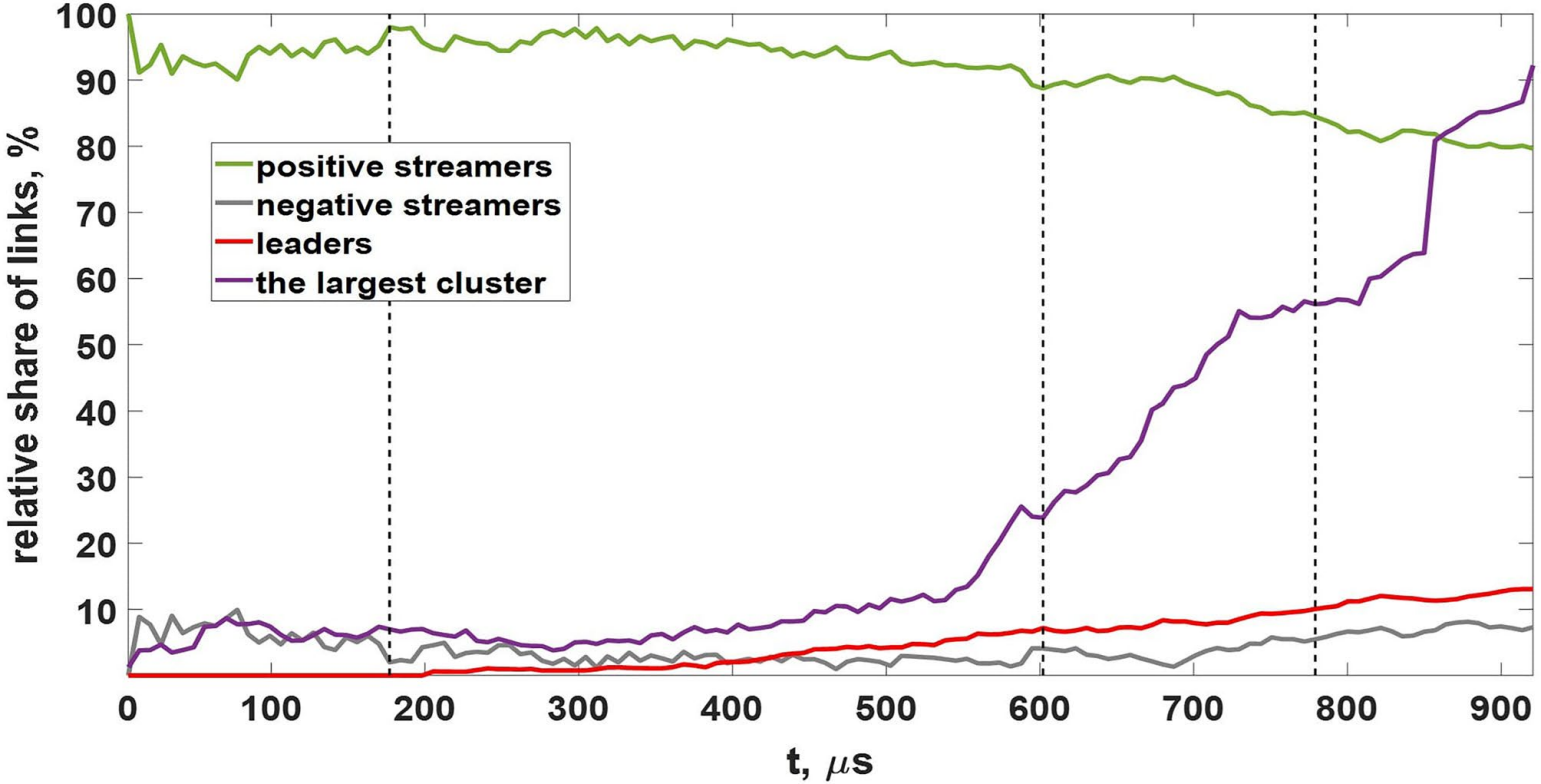
Dependence curves of the system composition in terms of positive and negative streamers and leaders (channel segments with conductivities $\sigma \ge 1$ S/m regardless of their polarities). Also shown the dynamics of the relative share of model links belonging to the main (the largest) cluster. Vertical dotted lines correspond to the moments of time shown in panels (**a–c**) in Fig. 10.

**Figure 13:**
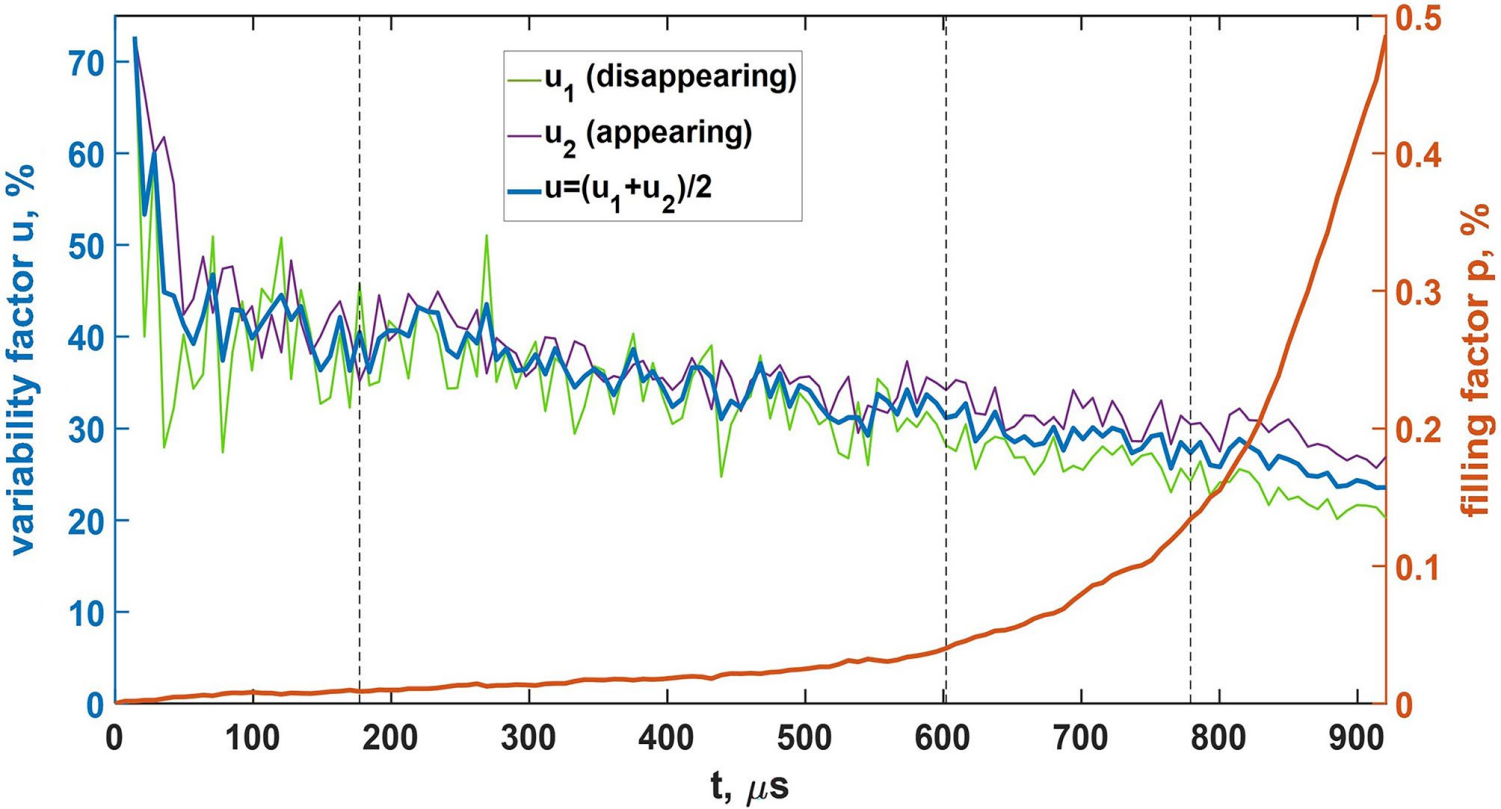
Temporal evolution of filling *p* and variability *u* factors reflecting dynamics of the self-organizing streamer/leader discharge system. Parameters $u_1$ and $u_2$ stand for the relative share of links disappearing and arising each time iteration, respectively, while the parameter *u* is their arithmetical mean. All the presented quantities are calculated not for the entire model volume, but for the horizontally reduced rectangular parallelepiped with $-20\le x[\text {m}]\le 20$ and $-20\le y[\text {m}]\le 10$, which includes the vast majority of model links during the entire simulation time (see Fig. 10). Vertical dotted lines correspond to the moments of time shown in panels (**a–c**) in Fig. 10.

At the purely streamer stage (the first 200 $\mu$s, see Fig. 12) the model dynamics is qualitatively similar to that of the basic model. Newly-formed streamer channels pick up charges left by the previously decayed ones. This way of charge transport is not effective because, unlike longer-lived EICRs, short-lived poorly-conducting streamer channels have no time to reach high conductivities and polarize which makes their contribution to the charge transport negligible. Situation drastically changes when first leader channels form inside several most developed streamer systems. Since well-conducting ($\sigma \ge 1$ S/m) leader sections can effectively polarize and amplify electric field at their tips, they provide conditions for further growth of discharge trees they belong to. There is a positive feedback loop between the measure of discharge structure polarization, which grows with increasing both conductivity and length, and the rate of its growth. It is important that bipolar leader channels electrostatically “feel” each other. As the electric field in the gap between the positive and negative parts of a pair of leaders is amplified, they gradually approach each other and merge. The ability of developed discharge trees to self-organize many times enhances effective conductivity of the medium. At this stage there is no need to have a big amount of more or less homogeneously distributed links (big filling factors) because internal system dynamics (cluster-cluster aggregation) makes them form a compact fractal chain that bridges the strong electric field region even with a relatively small number of links (see Fig. 10(d)). In particular, it follows from Fig. 13 that at the final stage, when a lightning leader channel extends from the bottom of the simulation domain to its top, the filling factor remains less than 0.5%.

**Figure 14:**
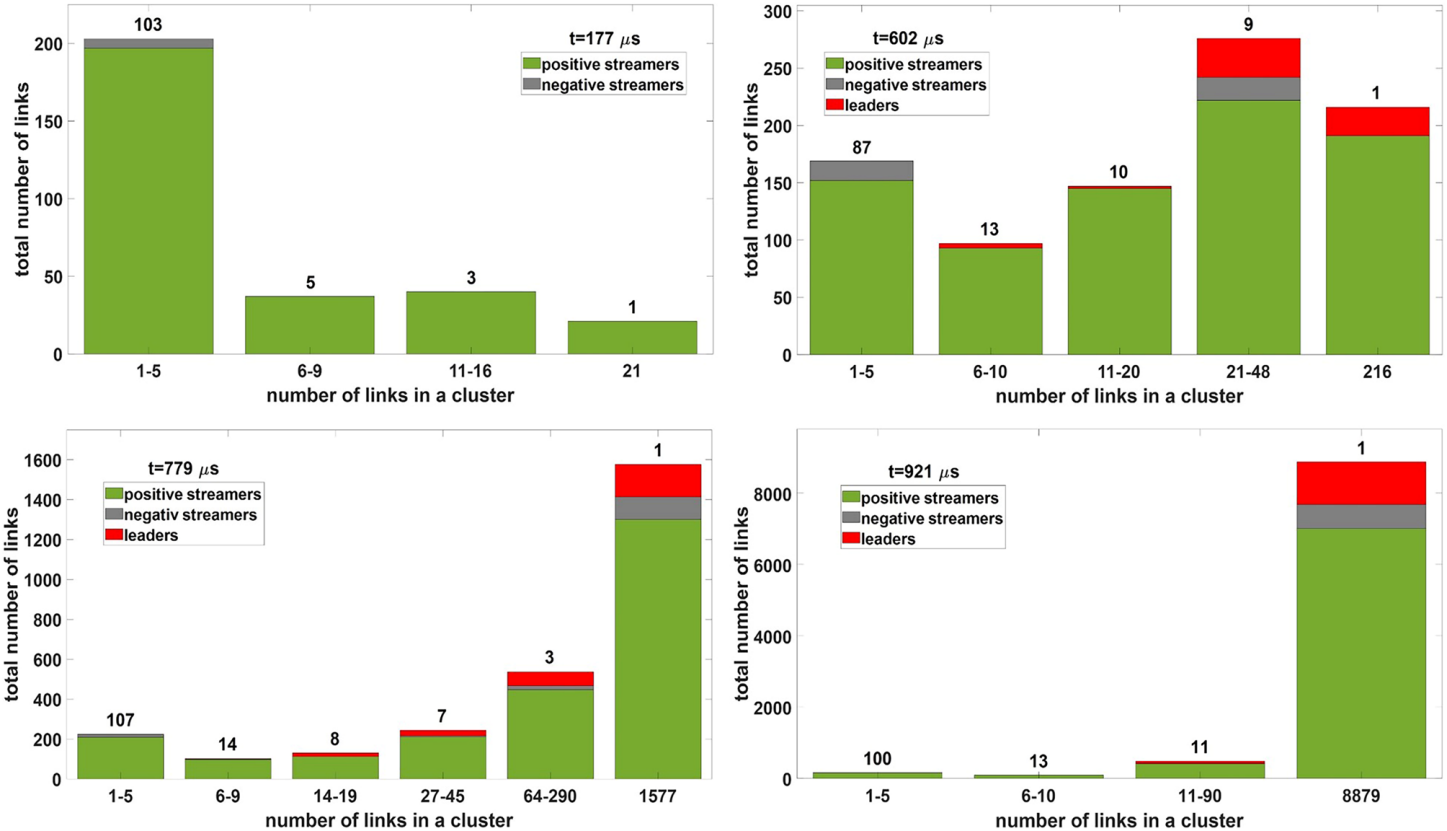
Distributions of model links by clusters of different sizes at four moments of time corresponding to panels (**a–d**) in Fig. 10. Except the most widespread positive streamers (green color), each bar may include some amount of negative streamers (grey color) and/or leaders (red color). Numbers at the tops of each bar denote amounts of clusters with the quantities of links shown on the horizontal axis. In each panel the biggest cluster is shown on the right.

The quantitative side of the system dynamics is presented in Figs. 12, 13, and 14. At the streamer stage the system consists of a nearly constant relatively small amount of continuously appearing and decaying streamer channels (mostly positive, see Fig. 12). Small discharge structures consisting of several streamer links cannot generate a leader channel. They either continue to grow or quickly die off. When several more “successful” streamer systems gain a critical amount (usually more than 10) of streamer links, polarization currents in their parent channels become sufficient to turn them into leader sections (compare the first ($t=177$ $\mu$s) and other panels in Fig. 14). Since the moment of appearance of first leader channels (approximately 200 $\mu$s from the start of simulation), survivability of discharge structures they belong to strongly increases. The phase of accelerating growth begins which is characterized by the rapidly increasing number of model links. Although the share of leader channels remains relatively small (less than 13%, see Fig. 12), their presence drastically changes the system dynamics in two aspects. Firstly, the areas of strong electric field at their tips ensure that the rate of new links appearance exceeds the rate of their decay (see the dynamics of quantities $u_1$, $u_2$, and *p* shown in Fig. 13). As a result, the number of model channels (primarily positive streamer links till the end of the simulation, see Fig. 12) rapidly increases. Secondly, electric field amplifications between positive and negative parts (streamer zones) of closely spaced bipolar leaders make them merge with each other. The process of cluster-cluster aggregation finally results in formation of the biggest discharge structure which “grabs” the most part (more than 90%, see Fig. 12) of model links. This tens of meters long well-polarized discharge tree can be considered as a self-sustaining lightning leader.

## Discussion

In this study, two consequently developing modes of relay charge transport inside an active part of a thundercloud were considered. Each of them was studied in the framework of a specially developed numerical model. At the initial pre-streamer stage the role of conductors is played by EICRs (elevated ionic conductivity regions) which are conducting plasma formations with characteristic sizes, lifetimes, and conductivities being of the order of 0.1–1 m, 1–10 s, and $10^{-10}-10^{-9}$ S/m, respectively^1^. Dynamics of EICRs results in multiscale electric field fluctuations (see Fig. 9), amplitudes of which increase up to the level when they produce numerous positive streamers. At the streamer/leader stage the mode of relay charge transport changes because streamer/leader systems are able to grow which facilitates their merger. This feature drastically increases transport ability of the self-organizing discharge system: there quickly arises a compact well-conductive cluster that bridges the “gap”.

Although a kind of self-organizing dynamics is also typical of the field-dependent second case of the basic approach (see  subsection “Simulation results (pre-streamer stage)”), in the advanced model the total number of streamer/leader channels (the filling factor *p*) and the relative share of disappearing/reappearing links (the variability factor *u*) are not fixed (see Fig. 13). This makes a very fast self-consistent formation of a long leader channel possible even in case of a relatively small total number of links. Note that a lightning seed shown in Fig. 10(d) plays a role of a percolating cluster in conditions when the filling factor $p\approx 0.5$% is about ten times smaller than the percolation threshold $p_c\approx 5$% obtained for the “classical” case of more or less homogeneous spatial distribution of links.

One more feature that distinguishes the basic model from the advanced one is that the latter has a threshold value of an ambient electric field under which there is no relay charge transport and, as a consequence, the formation of lightning channel does not occur. Although the probability formula (8) has no field threshold, it implies that there cannot be stable growth of even positive streamers in conditions of a too weak ambient electric field $E_0$. It was found that the minimum value of $E_0$, under which the system development stops at the stage of “streamer gas”, is about 55 kV/m at the considered 6 km altitude. As for the basic model, in the absence of any charge dissipation processes there is always a non-zero effective conductivity under $p>0$ and $u>0$. So, there are no threshold values of these quantities.

It is important to note that the basic model does not consider finiteness of the time of space charge existence. It is known that in conditions of a real thundercloud positive and negative ions attach to hydrometeors with characteristic frequency of loss $\nu _h$ being of the order of 0.1–1 s$^{-1}$ and are carried by the multiscale turbulent air flow^1^. This gradually destroys the areas of space charge concentration, model analogs of which are point charges at the spatial grid nodes. Because of this, predictions of the basic model may be imprecise for the values of *p* smaller than about 1% that correspond to field relaxation times bigger than 10 s or so (see Fig. 6).

As it was already mentioned, in the basic model it is assumed that both filling *p* and variability *u* factors are constant during the entire simulation time. This is because one of the goals of the study was to find out how relay conductivity $\sigma _{\text {eff}}$ varies with *p* for the two considered types of problem formulations (see subsection “Simulation results (pre-streamer stage)”). In a real situation, there must be a positive feedback loop that consists of the following stages: the growth of the spatio-temporal frequency of EICRs appearance, the rise of the level of space charge fluctuations, and the increase in the amplitudes of local electric field enhancements. As a result, in real physical systems the total number of conducting plasma formations as well as relay conductivity of the medium must gradually increase and the rate of their growth must accelerate in time (as it is in the advanced model, see Figs. 13 and 14). This process continues up to the moment when the magnitudes of electric field fluctuations become sufficient for initiation of positive streamers.

In the basic model, it is assumed that relay charge transport takes place only inside the simulation domain volume, i.e. the presence of EICRs (and any other conducting plasma formations) beyond its boundaries is neglected. Since it is expected that the rate of EICRs appearance increases with increasing electric field^1^, a natural equivalent of a simulation domain is an intracloud area of a relatively strong large-scale electric field that can also be called “an active part of a thundercloud”. The presence of non-zero relay conductivity inside these regions manifests itself in two ways. Firstly, it limits electric field growth by activation of relay mechanism of charge transport that extrudes strong electric field to the boundaries of a “conducting” area (see Fig. 8 for $0\le z \le 60$ m). The large-scale space charge redistribution creates new regions of a relatively strong electric field (see Fig. 8 for $z<0$ m and $z>60$ m) that get involved in relay charge transport as well. So, there may appear two oppositely directed waves of increased electric field that start from the borders of an active part of a thundercloud and propagate towards and against the large-scale electric field direction. Secondly, dynamics of alternating network of inhomogeneously distributed EICRs creates smaller-scale electric field enhancements that result in positive streamers initiation. Note that the magnitudes of localized electric field fluctuations increase with decreasing scale, so that they can easily exceed the values shown in Fig. 9 which were calculated in the framework of the model with the grid spacing of 1 m.

The abilities of alternating network of EICRs to limit large-scale electric field growth and to provide smaller-scale electric field enhancements ensure that conditions of seeding positive streamers appearance must be more or less stable (at least, they should not vary strongly). This is because they are regulated by the self-consistent thundercloud dynamics (relay charge transport). Although it is unknown what must be natural values of an ambient electric field and positive streamers volumetric density at the stage of “streamer gas”, there are some reasons to think that the advanced model correctly reproduces the main features of the lightning initiation process. Indeed, the used value of an ambient electric field $E_0$, set to 65 kV/m at the considered 6 km altitude (its ground-level equivalent is 133 kV/m), is lower than [3, Table 3.2] or at the lower boundary of [2, Table 3.1] maximum experimentally observed intracloud electric fields, which is expected for an active part of a thundercloud. As for the volumetric density of seeding streamers, it can hardly be very large because the spatio-temporal frequency of electric field amplifications, provided by EICRs and responsible for positive streamers initiation, is expected to increase gradually. Another reason is that, as simulation shows (see subsection “Simulation results (streamer/leader-based stage)”), even a negligibly small amount of primary positive streamers is enough to run the lightning leader formation process.

In the presented models, the grid spacing *a* was set to 1 m which coincides with characteristic sizes of both EICRs and first (seeding) positive streamer channels initiated by EICRs evolution. This is because a streamer length depends on the scale of electric field fluctuations which, in turn, is determined by the typical size of EICRs^1^. It should be noted that the form of dependence of relay conductivity $\sigma _{\text {eff}}$ on the filling factor *p* (see Eq. (15)) does not depend on the choice of both an ambient electric field magnitude $E_0$ and a model grid spacing *a*.

The advanced model assumes that discharge channels can also be considered as elements of alternating network of conducting links contributing to the relay charge transport which needs justification. First of all, it must be noted that a single streamer (ionization wave) consists of a poorly-conducting and quickly dissipating (due to recombination) plasma channel and a well-conducting streamer head which is responsible for ionization process^17^. Because of this, although a single streamer provides dipole-like charge separation, it cannot be considered as a perfectly (or even well) conducting channel. On the other hand, in a real thundercloud a streamer discharge is expected to have the form of branchy (and usually bipolar) systems which include a very big amount of simultaneously developing streamer channels (see, for example, studies^19–21^ and references therein). Multiple streamers passing through the same area of space provide gas heating which increases the conductivity of a “collective” channel^21^. It is well-known for a laboratory long spark where numerous streamers emanated by the leader tip create a so-called stem [14, par. 2.3.3]. The last is a short hot plasma formation in front of the leader channel that gradually turns into a new leader channel tip. Further, in a recent experiment^5^ with an artificial cloud of charged aerosol it was found that interaction of bipolar streamer systems results in the formation of a network containing hot plasma channels (unusual plasma formations) with parameters of plasma being intermediate between streamer and leader stages. In the advanced model, channels conductivities vary from a negligible initial value of $10^{-9}$ S/m to about 200 S/m typical of a hot leader channel (with the threshold level separating streamer channels from the leader ones being conditionally set to 1 S/m). For comparison, conductivities of 2 and 30 S/m correspond to equilibrium plasma temperatures of 4000 and 5000 K, respectively^22^. It is clear that “immature” streamer links can hardly play a role of conductors. But those of them that could survive long enough to raise their conductivities up to the leader level affect electric field distribution nearly as if they were perfectly conducting. As in the case of UPFs, the channels which concentrate bigger polarization currents first reach leader-like values of conductivities. In the model, they are sources (parent channels) of positive streamer systems or junctions between positive and negative parts of bipolar streamer systems at the moment of their merger. At later stages well-conducting cores of several streamer networks merge finishing lightning leader formation.

The advanced model does not take into account both microphysical (for example, the scattering and transport of electrons and ions) and gas dynamic (for example, thermal-ionizational instability and the appearance of shockwaves) aspects which are necessary to simulate the streamer-to-leader transition process. Instead of this, the model employs the simplified semi-empirical formula (17) to describe evolution of channels conductivity (temperature) from cold streamer to hot leader values. This limitation is necessary to reproduce the collective dynamics of thousands of simultaneously developing mutually interacting plasma channels (there are 9627 of them in panel (d) in Fig. 10) which is hardly possible for sophisticated models describing the dynamics of a single channel (e.g., studies^23–25^). The relevance of self-consistent charge transport models involving a large number of channels and capable to reproduce the macroscopic electrical properties of discharge system development was discussed by Luque and Ebert^26^ who simulated a branched positive streamer tree growing from an electrode. Note that the mentioned simplifications of the advanced model does not influence the conclusions of the study which rely on the macroscopic charge transport provided by a network of conducting channels.

## Summary

This study presents the analysis of the problem of charge transport in the framework of 3-D simple cubic lattice in the absence of a large-scale conducting cluster bridging plates of a conditional capacitor. Model results were used to find analytical forms of dependencies of effective conductivity of the medium on the relative share of conducting elements for both field-dependent and field-independent modes of model links orientations. This type of charge transport (conductivity) can be called “relay” because a large-scale separation of point charges is provided by stochastic dynamics of separated clusters of conducting links. Although these clusters are not connected in snapshots of the system, they form a continuous chain in 4-D spatio-temporal continuum as the clusters have alternating geometry. As a result, in the 3-D space charges left by the decayed clusters are picked up and further transferred by the newly-formed ones.

Two numerical models were used to consider pre-streamer and streamer/leader-based modes of relay charge transport. The first mechanism relies on continuously arising and dissipating elevated ionic conductivity regions (EICRs) which are conducting plasma formations with characteristic scales, lifetimes, and conductivities of the order of 0.1–1 m, 1–10 s, and $10^{-10}-10^{-9}$ S/m, respectively^1^. Their dynamics creates electric field fluctuations with progressively increasing magnitudes which eventually provides positive streamers initiation. At the streamer/leader stage the effectiveness of relay charge transport rapidly increases because of abilities of discharge channels to grow and merge with each other. In fact, it means that at the streamer/leader phase the system dynamics (relay charge transport) starts to reproduce itself at progressively larger spatial scales. Accelerated coalescence of self-organizing streamer/leader systems finally results in formation of a percolating cluster bridging an active part of a thundercloud. The fact that the resulting compact (fractal) well-conducting channel cluster can be associated with a self-sustaining lightning seed accentuates the role of relay charge transport in the lightning initiation process.

The main findings of the study are the following:

1. Alternating network of a relatively small fraction of conducting plasma formations (EICRs or streamer/leader channels) can provide relay charge transport inside a thundercloud even in the absence of a conducting channel bridging the “gap” (a region of space initially occupied by a relatively strong large-scale electric field).
2. Relay conductivity is a sharply increasing function of the relative share of space occupied by conducting plasma formations.
3. Internal system dynamics ensures that the value of relay conductivity increases in time as well as the rate of its growth.
4. Effectiveness of relay charge transport strongly increases if both locations and orientations of reappearing conducting plasma formations are determined by the local electric field distribution which makes the system dynamics self-organizing.
5. At the initial (much more longer and more homogeneous) stage relay charge transport is provided by an alternating network of elevated ionic conductivity regions that prepare conditions for positive streamers inception.
6. At the second streamer/leader phase self-organizing mode of relay charge transport repeats itself at progressively larger spatial scales ending with a self-sustaining lightning leader formation.

## Methods

This section presents the scheme of numerical calculations used in the basic (pre-streamer) model which is described in subsection “Pre-streamer model description”.

The simulated system consists of a set of perfectly conducting links connecting the neighboring nodes of a simple cubic lattice. These links are grouped into clusters of different sizes with the minimal cluster being a single isolated link (see Figs. 2, 3, and 5). Since the clusters are placed in the area occupied by a non-zero large-scale electric field ${\varvec{E}}_0$, they polarize redistributing point charges in their nodes. Each time iteration a certain part of randomly chosen links disappears and simultaneously reappears between another (also randomly chosen) places causing stochastic charge transport. Let’s assume that after another act of model links redistribution the system consists of $N_{cl}$ perfectly conducting clusters each of which includes $N_i\ge 2$ ($i=1:N_{cl}$) spatial grid nodes. Besides, there are $N_{ext}$ additional point charges that were left by the previously decayed clusters but do not belong to any presently existing conducting structure. As geometry of the network of channels has changed, one must recalculate point charges at spatial grid nodes belonging to clusters. The result must meet the conditions (2) and (3) which ensure clusters equipotentiality and charge conservation law, respectively. To achieve the required result, the following system of linear algebraic equations must be solved:

19 $$\begin{aligned} {\left\{ \begin{array}{ll} \underbrace{\frac{1}{4\pi \varepsilon _0}\left( \overbrace{\frac{q_1^{1}}{(a/2)} + ... + \frac{q_k^{1}}{|{\varvec{r}}_k^1-{\varvec{r}}_1^1|} + ... + \frac{q_{N_1}^{1}}{|{\varvec{r}}_{N_1}^1-{\varvec{r}}_1^1|}}^{N_1 \text {charges belonging to the cluster } 1} + \sum _{i=2}^{N_{cl}}\sum _{j=1}^{N_{i}}\frac{q_j^i}{|{\varvec{r}}_j^i-{\varvec{r}}_1^1|}\right) - \varphi _1 = -\frac{1}{4\pi \varepsilon _0}\sum _{i=1}^{N_{\text {ext}}}\frac{q_i^{\text {ext}}}{|{\varvec{r}}_i^{\text {ext}}-{\varvec{r}}_1^1|} + ({\varvec{E}}_0 \cdot {\varvec{r}}_1^1)}_{\text {line for the node } {\varvec{r}}_1^1 \text { of the cluster 1}} &{} \\ ... &{} \\ \underbrace{\frac{1}{4\pi \varepsilon _0}\left( \sum _{i=1}^{l-1}\sum _{j=1}^{N_{i}}\frac{q_j^i}{|{\varvec{r}}_j^i-{\varvec{r}}_k^l|} + \overbrace{\frac{q_1^{l}}{|{\varvec{r}}_1^l-{\varvec{r}}_k^l|} + ... + \frac{q_k^{l}}{(a/2)} + ... + \frac{q_{N_l}^{l}}{|{\varvec{r}}_{N_l}^l-{\varvec{r}}_k^l|}}^{N_l \text {charges belonging to the cluster } l} + \sum _{i=l+1}^{N_{cl}}\sum _{j=1}^{N_{i}}\frac{q_j^i}{|{\varvec{r}}_j^i-{\varvec{r}}_k^l|}\right) - \varphi _l = -\frac{1}{4\pi \varepsilon _0}\sum _{i=1}^{N_{\text {ext}}}\frac{q_i^{\text {ext}}}{|{\varvec{r}}_i^{\text {ext}}-{\varvec{r}}_k^l|} + ({\varvec{E}}_0 \cdot {\varvec{r}}_k^l)}_{\text {line for the node } {\varvec{r}}_{k}^l \text { of the cluster } l} &{} \\ ... &{} \\ \underbrace{\frac{1}{4\pi \varepsilon _0}\left( \sum _{i=1}^{N_{cl}-1}\sum _{j=1}^{N_{i}}\frac{q_j^i}{|{\varvec{r}}_j^i-{\varvec{r}}_{N_{N_{cl}}}^{N_{cl}}|} + \overbrace{\frac{q_1^{N_{cl}}}{|{\varvec{r}}_1^{N_{cl}}-{\varvec{r}}_{N_{N_{cl}}}^{N_{cl}}|} + ... + \frac{q_{k}^{N_{cl}}}{|{\varvec{r}}_{k}^{N_{cl}}-{\varvec{r}}_{N_{N_{cl}}}^{N_{cl}}|} + ... + \frac{q_{N_{N_{cl}}}^{N_{cl}}}{(a/2)}}^{N_{N_{cl}} \text {charges belonging to the cluster } N_{cl}}\right) - \varphi _{N_{cl}} = -\frac{1}{4\pi \varepsilon _0}\sum _{i=1}^{N_{\text {ext}}}\frac{q_i^{\text {ext}}}{|{\varvec{r}}_i^{\text {ext}}-{\varvec{r}}_{N_{N_{cl}}}^{N_{cl}}|} + ({\varvec{E}}_0 \cdot {\varvec{r}}_{N_{N_{cl}}}^{N_{cl}})}_{\text {line for the node } {\varvec{r}}_{N_{N_{cl}}}^{N_{cl}} \text { of the cluster } N_{cl}} &{} \\ \underbrace{q_1^1 + ... + q_k^1 + ... + q_{N_1}^1 = q_1^{'1} + ... + q_k^{'1} + ... + q_{N_1}^{'1}}_{\text {charge conservation law for the cluster 1}} &{} \\ ... &{} \\ \underbrace{q_1^l + ... + q_k^l + ... + q_{N_l}^l = q_1^{'l} + ... + q_k^{'l} + ... + q_{N_l}^{'l}}_{\text {charge conservation law for the cluster } l} &{} \\ ... &{} \\ \underbrace{q_1^{N_{cl}} + ... + q_k^{N_{cl}} + ... + q_{N_{N_{cl}}}^{N_{cl}} = q_1^{'N_{cl}} + ... + q_k^{'N_{cl}} + ... + q_{N_{N_{cl}}}^{'N_{cl}}}_{\text {charge conservation law for the cluster } N_{cl}}. &{} \\ \end{array}\right. } \end{aligned}$$

In the system of equations (19) superscripts and subscripts of charges $q_j^i$ and radius-vectors ${\varvec{r}}_j^i$ designate the number of a cluster $i=1:N_{cl}$ and the number of a node $j=1:N_i$ from the cluster *i*, respectively. The subscripts of potentials $\varphi _i$ stand for numbers of equipotential clusters. Point charges and radius-vectors for nodes that do not belong to any cluster have the superscript “ext” (from the word “external”). The first $\sum _{i=1}^{N_{cl}}N_i$ lines of the system correspond to Eq. (1) formulated for all the nodes belonging to conducting clusters, while the last $N_{cl}$ lines present Eq. (3) formulated for each particular cluster. Note that $q_j^{'i}$ are charges located at the nodes of a cluster *i* before the recalculation procedure which are to be redistributed over the cluster by potential equalization currents. As the full system of equations (19) has $\sum _{i=1}^{N_{cl}}N_i+N_{cl}$ lines, only some of them can be shown. It is convenient to represent the system of equations (19) in the matrix form:

20 $$\begin{aligned} {\hat{A}} \cdot X = B. \end{aligned}$$

The matrix ${\hat{A}}$ can be divided into four blocks:

21 $$\begin{aligned} {\hat{A}} = \begin{pmatrix} {\hat{C}}_{\sum _{i=1}^{N_{cl}}N_i \text { by } \sum _{i=1}^{N_{cl}}N_i} &{} {\hat{D}}_{\sum _{i=1}^{N_{cl}}N_i \text { by } N_{cl}} \\ {\hat{E}}_{N_{cl} \text { by } \sum _{i=1}^{N_{cl}}N_i} &{} {\hat{O}}_{N_{cl} \text { by } N_{cl}} \end{pmatrix}. \end{aligned}$$

The biggest block ${\hat{C}}$ consists of $N_{cl}\times N_{cl}$ sections:

22 $$\begin{aligned} {\hat{C}}= \begin{pmatrix} {\hat{c}}_{1,1} &{} ... &{} {\hat{c}}_{1,k} &{} ... &{} {\hat{c}}_{1,N_{cl}} \\ &{} &{} ... &{} &{} \\ {\hat{c}}_{k,1} &{} ... &{} {\hat{c}}_{k,k} &{} ... &{} {\hat{c}}_{k,N_{cl}} \\ &{} &{} ... &{} &{} \\ {\hat{c}}_{N_{cl},1} &{} ... &{} {\hat{c}}_{N_{cl},k} &{} ... &{} {\hat{c}}_{N_{cl},N_{cl}} \end{pmatrix}. \end{aligned}$$

Submatrixes ${\hat{c}}_{s,k}$ have dimensions $N_s\times N_k$ and consist of elements

23 $$\begin{aligned} \left\{ {\hat{c}}_{s,k}\right\} _{i,j}= {\left\{ \begin{array}{ll} \frac{1}{|{\varvec{r}}_j^k-{\varvec{r}}_i^s|}, &{} \text {if } {\varvec{r}}_j^k\ne {\varvec{r}}_i^s \\ \frac{1}{(a/2)}, &{} \text {otherwise.} \end{array}\right. } \end{aligned}$$

As for the other components of the matrix ${\hat{A}}$,

24 $$\begin{aligned} D_{k,i}= {\left\{ \begin{array}{ll} -1, &{} \text {if } \sum _{s=1}^{i-1}N_s +1\le k \le \sum _{s=1}^{i}N_s \\ 0, &{} \text {otherwise,} \end{array}\right. } \end{aligned}$$

25 $$\begin{aligned} E_{i,k}= {\left\{ \begin{array}{ll} 1, &{} \text {if } \sum _{s=1}^{i-1}N_s +1\le k \le \sum _{s=1}^{i}N_s \\ 0, &{} \text {otherwise,} \end{array}\right. } \end{aligned}$$

and ${\hat{O}}$ is the zero matrix. The elements of the right hand side of the system (20) are

26 $$\begin{aligned} {\left\{ \begin{array}{ll} B_{\sum _{s=1}^{i-1}N_s+j \text { } (1\le j\le N_i)} = -\frac{1}{4\pi \varepsilon _0}\sum _{k=1}^{N_{\text {ext}}}\frac{q_k^{\text {ext}}}{|{\varvec{r}}_k^{\text {ext}}-{\varvec{r}}_j^i|} + ({\varvec{E}}_0 \cdot {\varvec{r}}_j^i), &{} \text {if [the line number]} \le \sum _{s=1}^{N_{cl}}N_s \\ B_{\sum _{s=1}^{N_{cl}}N_s+i \text { } (1\le i\le N_{cl})} =\frac{1}{4\pi \varepsilon _0}\sum _{k=1}^{N_i}q_k^{'i}, &{} \text {if } \sum _{s=1}^{N_{cl}}N_s<\text {[the line number]} \le \sum _{s=1}^{N_{cl}}N_s+N_{cl}. \end{array}\right. } \end{aligned}$$

The vector of unknowns has the following components:

27 $$\begin{aligned} {\left\{ \begin{array}{ll} X_{\sum _{s=1}^{i-1}N_s+j \text { } (1\le j\le N_i)} = \frac{q_j^i}{4\pi \varepsilon _0}, &{} \text {if [the line number]} \le \sum _{s=1}^{N_{cl}}N_s \\ X_{\sum _{s=1}^{N_{cl}}N_s+i \text { } (1\le i\le N_{cl})} =\varphi _i, &{} \text {if } \sum _{s=1}^{N_{cl}}N_s<\text {[the line number]} \le \sum _{s=1}^{N_{cl}}N_s+N_{cl}. \end{array}\right. } \end{aligned}$$

It includes the sought point charges multiplied by $\frac{1}{4\pi \varepsilon _0}$ and potentials of the clusters. As the absolute values of charges $q_j^i$ are many orders of magnitude lower than those of potentials $\varphi _i$, the factor $\frac{1}{4\pi \varepsilon _0}$ before $q_j^i$ is needed to improve the accuracy of calculations. Finally, one can find the updated charges $q_j^i$ at the nodes of conducting clusters via the following formula

28 $$\begin{aligned} q_j^i=4\pi \varepsilon _0\left( {\hat{A}}^{-1}B\right) _{\sum _{s=1}^{i-1}N_s+j \text { } (1\le j\le N_i)}. \end{aligned}$$

The knowledge of these charges allows one to find the mean electric potentials $U(z_u)$ and $U(z_d)$ at the levels of $z_u=48$ m and $z_d$=12 m in order to obtain an averaged electric field $E_{\text {eff}}$ in the inner part of the system (see Eq. (7)) which is needed for relay conductivity calculation (see paragraph “Model equations”).

## Data Availability

A movie visualizing the lightning “seed” formation, several snapshots of which are shown in Fig. 10, is available online at https://zenodo.org/record/4319886#.X9Y5lODVLIU4.

## Abbreviations

EICRs: Elevated ionic conductivity regions
CPFs: Conductive plasma formations
UPFs: Unusual plasma formations
